# Towards high-accuracy bacterial taxonomy identification using phenotypic single-cell Raman spectroscopy data

**DOI:** 10.1093/ismeco/ycaf015

**Published:** 2025-03-09

**Authors:** Guangyu Li, Zijian Wang, Chieh Wu, Dongqi Wang, Il Han, Jangho Lee, David R Kaeli, Jennifer G Dy, Kilian Q Weinberger, April Z Gu

**Affiliations:** School of Civil and Environmental Engineering, Cornell University, Ithaca, NY 14850, United States; Department of Biological and Environmental Engineering, College of Agriculture and Life Sciences, Cornell University, NY 14850, United States; Center for Research on Programmable Plant Systems, 103 Rice Hall, Cornell University, Ithaca, NY 14850, United States; Department of Electrical and Computer Engineering, Northeastern University, Boston, MA 02115, United States; Department of Municipal and Environmental Engineering, School of Water Resources and Hydro-Electric Engineering, Xi’an University of Technology, Xi’an, Shaanxi, 710021, China; School of Civil and Environmental Engineering, Cornell University, Ithaca, NY 14850, United States; School of Civil and Environmental Engineering, Cornell University, Ithaca, NY 14850, United States; Department of Electrical and Computer Engineering, Northeastern University, Boston, MA 02115, United States; Department of Electrical and Computer Engineering, Northeastern University, Boston, MA 02115, United States; Department of Computer Science, Cornell University, Ithaca, NY 14850, United States; School of Civil and Environmental Engineering, Cornell University, Ithaca, NY 14850, United States; Center for Research on Programmable Plant Systems, 103 Rice Hall, Cornell University, Ithaca, NY 14850, United States

**Keywords:** single-cell Raman spectroscopy, machine learning, algorithm benchmark, single-cell taxonomy identification, microbial ecology, environmental microbiome

## Abstract

Single-cell Raman Spectroscopy (SCRS) emerges as a promising tool for single-cell phenotyping in environmental ecological studies, offering non-intrusive, high-resolution, and high-throughput capabilities. In this study, we obtained a large and the first comprehensive SCRS dataset that captured phenotypic variations with cell growth status for 36 microbial strains, and we compared and optimized analysis techniques and classifiers for SCRS-based taxonomy identification. First, we benchmarked five dimensionality reduction (DR) methods, 10 classifiers, and the impact of cell growth variances using a SCRS dataset with both taxonomy and cellular growth stage labels. Unsupervised DR methods and non-neural network classifiers are recommended for at a balance between accuracy and time efficiency, achieved up to 96.1% taxonomy classification accuracy. Second, accuracy variances caused by cellular growth variance (<2.9% difference) was found less than the influence from model selection (up to 41.4% difference). Remarkably, simultaneous high accuracy in growth stage classification (93.3%) and taxonomy classification (94%) were achievable using an innovative two-step classifier model. Third, this study is the first to successfully apply models trained on pure culture SCRS data to achieve taxonomic identification of microbes in environmental samples at an accuracy of 79%, and with validation via Raman-FISH (fluorescence *in situ* hybridization). This study paves the groundwork for standardizing SCRS-based biotechnologies in single-cell phenotyping and taxonomic classification beyond laboratory pure culture to real environmental microorganisms and promises advances in SCRS applications for elucidating organismal functions, ecological adaptability, and environmental interactions.

## Introduction

Single-cell Raman spectroscopy (SCRS) is an emerging phenotyping biotechnology for biochemical fingerprinting and quantification by analyzing the non-elastic Raman scattering of organisms, cells, or other biomaterials [1]. SCRS provides a cultivation-free and non-invasive way to identify cell phenotypes [2], analyze metabolic state [3], and quantify biochemical compositions [4]. In advance to other single-cell biotechniques such as fluorescence-activated cell sorting and single-cell RNA sequencing, SCRS enables real-time monitoring of living cell activity [5–7], phenotype-based cell sorting [8], rapid cell, or virus identification [9–11], thus promising advanced applications in environmental, microbiological, and ecological studies [12].

Recent advances have applied SCRS to study microbial ecology for a detailed understanding of microbial diversity, interactions, and functions within ecosystems. Environmental microbiomes are particularly complex due to the presence of unknown taxa, varying growth stages, and diverse metabolic statuses [13–15]. For instance, SCRS with heavy water revealed active phosphorus solubilizing bacteria have significant difference in abundance and *in situ* function across soil types [16], and SCRS-based sorting platform has revealed the active bacteria with mucin degradation in the mouse colon [12]. Traditional methods, often reliant on cultivation or genetic sequencing, can be time-consuming, labor-intensive, and sometimes biased due to selective growth conditions. SCRS, with its ability to provide real-time, non-invasive, and cultivation-free analysis, offers a transformative approach to microbial ecology. Equipped with machine learning (ML), SCRS-based methods go beyond rapid identification of microbial taxa by simultaneously providing phenotypic characterization and identifications of taxonomy, growth stages, etc. [7, 9, 17]. This dual capability enhances our understanding of the structure and function of complex microbial communities, facilitating more comprehensive and dynamic ecological and single-cell studies [18, 19].

SCRS-based organism taxonomy identification is of great interest for advancing microbial ecology investigations. Further, it provides insights into the fundamental biology question regarding the relationships between taxonomic biodiversity and functional diversity [20–22]. Six most previous studies chose the algorithms at per-lab preference, and no algorithmic benchmarking of combinations of dimension reduction and classifiers was evaluated. Furthermore, comparison between traditional classifiers and neural networks is missing, specifically considering when the available dataset size could be suboptimal for deep learning models [23] due to labor intensiveness [24]. Thus, algorithm selections can be a critical gap for developing standardized SCRS-based taxonomy identification that allows large-scale comparative analyses of novel biological discoveries across different labs and domains.

One of the major challenges in SCRS-based taxonomic identification is the high susceptibility of the SCRS signal to both systematic variations (i.e. Raman equipment, experimental protocols) and biological conditions (i.e. growth stage, nutrients conditions, etc.). A limited number of studies on SCRS-based microbial strain/virus identification exploration showed good accuracy (> 97%) with relatively small datasets of organism/cells with less phylogenetic diversity and growth condition variations (200–2000 of Raman spectrum) [10, 25], and lower (75–95%) accuracy for larger datasets that contain data from wider range of sources and thus inherent more system or conditional noises (> 10 000 Raman spectrum) [9, 24]. However, to our best knowledge, there is no study about the impact of biological growth stage on SCRS-based taxonomy identification although these factors can significantly shift cell’s metabolic activity, gene expression patterns, and molecular signaling pathways [26]. These factors are especially significant in microbial ecology because environmental samples exhibit high variability and complexity in growth stages and nutrient conditions, which influences the reliability of SCRS-based taxonomy identification and biochemical fingerprinting [27–29]. Whether accurate SCRS-based taxonomy identification can be achieved with the uncontrollable biological variances from cell growth stage or conditions remains a crucial yet unexplored question for various SCRS applications [30].

In this study, we aim to address three major questions to contribute to the advances of microbial ecology applications of ML-enabled SCRS: (i) What is the algorithmic benchmarking needed to achieve high taxonomy identification accuracy on lab-cultured strains, given the difficulty to obtain and the lack of obligate reference databases for environmental microbiomes with simultaneous Raman and strain-level taxonomy labels? (ii) How do biological variables, such as cellular growth stage, impact the identification accuracy on lab-cultured strains, given that environmental samples do not have growth stage information? (iii) Can common ML algorithms, trained with laboratory pure cultures, be applied to real environmental cell data, considering the lack of corresponding datasets and algorithmic benchmarks in previous studies? To address these questions, we obtained the largest duo-labeled (taxonomic identity and growth stage) SCRS dataset to date, comprising 17 208 spectra from 36 soil and aqueous bacterial strains at four growth stages. Firstly, we benchmarked various dimension reduction and classifier combinations for accurate taxonomy identification (workflow summarized in Supplementary Fig. S1), finding those certain combinations significantly outperformed others, achieving taxonomy identification accuracy of over 96% for lab-cultured strains without accounting for growth stage information (the T-only approach). Secondly, we investigated the impact of different cell growth stages on the accuracy of SCRS-based taxonomy identification. Incorporating growth stage information into the models (the T/G approach, read as T-on-G) improved taxonomy identification accuracy by up to 3%. Conversely, when growth stage information was not incorporated, taxonomy accuracy deteriorated due to feature overlap in a two-step method that first predicts the growth stage and then identifies the taxonomy (the G-T approach). Lastly, we tested the applicability of our trained model on real environmental samples, demonstrating robust identification performance despite the complexity and variability of these samples. This study advances the standardization of ML-enabled SCRS applications in microbial ecology, enhancing the ability to simultaneously and accurately characterize microbial communities and their functions in complex ecosystems.

## Materials and methods

### SCRS dataset of 36 bacterial strains

The dataset contains 17, 208 spectra of 36 microorganism strains that commonly present in water and soil environment with functions of plant health, symbiosis, and bioremediation (strains/species info shown in Supplementary Table S1). These 36 strains belong to four phyla and 16 genera, being chosen to benchmark algorithmic capability to identify organisms at different taxonomic level, such as genus, strains, etc. Each strain was individually cultivated and SCRS were taken when the cells were in exponential phase and at the beginning, mid-point, and end of the stationary phase (Supplementary Text S1). We sampled the stationary phase with higher resolution since most of bacteria are in stationary phase in natural environment, which is typically oligotrophic [31]. All spectra were firstly preprocessed using the same protocol [30, 32–35] (including background subtraction, smoothing, and baseline correction) before DR and classification analysis. More than 100 SCRS spectra was collected per strain per sampling time points, which is sufficient for pure cultures in leu of the statistical analysis of minimal numbers of cells as detailed by Li et al. [36]. More statistical and detailed SCRS experimental parameters can be found in Supplementary Text S1 & Tables S2–S8.

### SCRS test dataset of real environmental samples

To evaluate whether the models trained with pure cultures library can be applied to identify the bacterial taxonomy in real environmental samples, a case test dataset was collected by Raman-FISH, where 16 s rRNA-based FISH probes target specific taxonomic groups [37, 38] (Details in Supplementary Text S1). The case study is focused on SCRS-based identification of agent genera *Comamonas* and *Tetrasphaera* in wastewater enhanced phosphorus removal ecosystem, sampled from Calumet Wastewater Treatment Facility (Calumet, IL, USA). SCRS data for model training were collected for two strains *Tetrasphaera elongata* (DSM 14184) and *C. testosterone* (ATCC 11996) at laboratory conditions as shown in Supplementary Text S1. Two case studies were tested: (i) SCRS-based taxonomic identification of *Comamonas* and *Tetrasphaera* using model trained with SCRS data of only these two pure-culture strains, and (ii) using model trained with SCRS data of combined 36 total pure-culture strains by extending the reference database to include culture from varying growth conditions, mimicking the real environmental applications. The extended training dataset was created by merging the *T. elongata* and *C. testosteroni* pure culture spectra in the 36-strain dataset, then re-balancing at genus-level to match the outputs. The final extended training dataset contained 18 genera labels with 100 spectra each. The 100 SCRS spectra for each of the genus from the wastewater treatment were obtained with taxonomic labels identified by Raman-FISH (Supplementary Text S1).

### Dimensionality reduction

In this study, we tested the SCRS spectra classification performance with a combination of different DR methods and classifiers. We chose both commonly used novel algorithms to cover categories of unsupervised/supervised and linear/nonlinear DR methods. Supervised DR algorithms take label information and optimize for a reduced number of features that can distinguish those labels. Unsupervised methods do not utilize label information to establish taxonomy-phenotype linkages, while maximizing the preservation of information to represent the entire dataset in a low-dimensionality space. Linear DR transforms the dataset based on linear combinations of the input features, and nonlinear DR does otherwise. Besides the DR methods listed below, the method referred to as “None” in this study means the classifiers were trained with full dimensionality without reduction. DR algorithms are summarized in Table 1a and Supplementary Text S2 for additional details.

**Table 1a:** Summary of dimensionality reduction (DR) methods.

**Abbreviation**	**Full Name/Description**	**Transformation linearity**	**Supervised (S)/unsupervised (N)**
None	no DR performed / identical	yes	N
ISM_SDR	iterative spectrum method [39]	no	S
PCA	canonical principal component analysis [40]	yes	N
LDA	linear discriminant analysis [40]	yes	S
KPCA	kernel (radial basis function) version of PCA [41]	no	N
SUP_PCA	supervised PCA [42]	yes	S

### Classifiers

A variety of classifiers were tested in this study to evaluate their performance with the combination of previously listed DR methods. In general, these models can be categorized into two types: linear and nonlinear. The category of linear/nonlinear classifiers are defined as if models feature a linear/nonlinear decision boundary [40]. In comparison, nonlinear classifiers are more flexible to approximate complex decision boundaries at a cost of higher potential of overfitting without cautious regularization [40]. Classifier algorithms are summarized in Table 1b and Supplementary Text S3 for additional details.

**Table 1b:** Summary of classifiers.

**Abbreviation**	**Full Name/Description**	**Linearity**
GNB	Gaussian naive Bayes classifier [40]	generalized linear
KNN	K-nearest neighbor classifier (K = 10) [43]	no
LDA	linear discriminant analysis [40]	linear
LR	logistic regression [43]	generalized linear
RF	random forest (100 decision trees) [40]	no
SVM_LIN	linear support vector machine [44]	yes
SVM_LIN_CV	linear support vector machine with regularization parameter optimized by cross-validation [44]	yes
SVM_RBF	kernel (radial basis function) support vector machine [40]	no
SVM_RBF_CV	kernel (radial basis function) support vector machine with regularization parameter and RBF kernel scaling factor (σ) optimized by cross-validation [40]	no
NN	neural network, Multi-Layer Perceptron (MLP) in this study.	no

### Growth-stage-then-taxonomy 2-step classification ensemble

A 2-step classifier ensemble with growth stage and taxonomy classifications in sequence (G-T model) was developed to test the impact on taxonomy identification accuracy after recovering growth stage information when it is not available as prior knowledge. The 2-step ensemble first predicts a testing sample of its growth stage (Exp, S1, S2, or S3), called G-step, then the taxonomy, called T-step, using models specifically trained for each predicted label from the G-step. The G-step and the T-step models have independent DR and classifier setups.

### Cross-validation

In our study, cross-validation is used for both model accuracy evaluation and model hyperparameter optimization. Since each cross-validation will address only one of the above two purposes, multiple independent cross-validations will be performed for models that require both accuracy evaluation and hyperparameter optimization.

#### CV in accuracy evaluation

It is a convention to evaluate classifier performance with samples aside from training data to prevent potential overestimation. CV is a common technique for this purpose, which partitions the whole dataset into multiple splits, using nine of them for training and the last for evaluation. A full 10-fold CV will partition the dataset into 10 splits and repeat the above process 10 times until each split is evaluated. The final model accuracy is a summarization of these 10 evaluation results.

#### CV in hyperparameter optimization

If a model needs hyperparameter optimization, an independent optimization CV is incorporated into each iteration of the evaluation CV. The optimization CV combines and re-splits the nine splits of training data from the evaluation CV, while the testing split of the evaluation CV is untouched. The hyperparameters are tuned by grid search, i.e. testing a list of candidate parameter values and then picking the one yielding the highest accuracy.

### Accuracy as the performance metric

In this study, accuracy is used to evaluate model performances. Classification accuracy is calculated as the proportion of testing samples that are assigned with correct labels among all testing samples, varying between 0.0 (0%) and 1.0 (100%). This measure represents the proportion of cells whose taxonomy labels are correctly recognized, therefore is an important evaluation of model performance at the microbial community level in SCRS classification applications. One disadvantage of using accuracy as a performance metric is its high bias toward the majority class in imbalanced training data (e.g. 99% of samples belong to a single class). In such cases, using accuracy will be difficult to disqualify a model classifying everything as the majority class, and metrics that are more sensitive to false positives (e.g. area under the receiver operating characteristic curve (AUC under ROC) or F1 score) are necessary. Our dataset is not subject to this failure, as all 36 strains are proportioned between 0.7% and 3.6%, without a majority class to cause potential bias introduced by accuracy.

The DR and classifiers used in this study are summarized in Table 1a and 1b, respectively. All the algorithms for DR and classifiers were achieved in Python using the package scikit-learn v0.19, PyTorch v1.13.0 (with NVIDIA CUDA 11.7 support), open source libraries (https://github.com/albat3ross/ISM_supervised_DR.git, https://github.com/endsley/wuML.git), or custom scripts. All GPU-related workload was performed on cluster nodes equipped with NVIDIA V100 Tensor Core GPU running CentOS 7.

### Model accuracy comparison and null-hypothesis significance test

To assess if the differences in model accuracies are statistically significant, Bayesian estimation supersedes the t-test (BEST) [45] was conducted using Metropolis-Hastings algorithm [46], a Markov Chain Monte-Carlo (MCMC) sampler. Unlike the t-test, BEST does not assume data normality and can statistically accept the null hypothesis rather than only a rejection [45]. The mean and standard deviation used to model each model’s accuracy distributions are assumed to have normal prior distributions. The first 1, 000 iterations of the MCMC process were considered and manually validated as the “burn-in” phase and were excluded from p-value calculations. The p-value was calculated as the probability where the null hypothesis is true in the MCMC sampling results. If multiple significance null-hypothesis tests are conducted simultaneously, the false discovery rate is controlled to reduce false positives [47].

## Results

### SCRS-based taxonomic benchmarking performance without growth stage labels (T-only)

**Figure 1:**
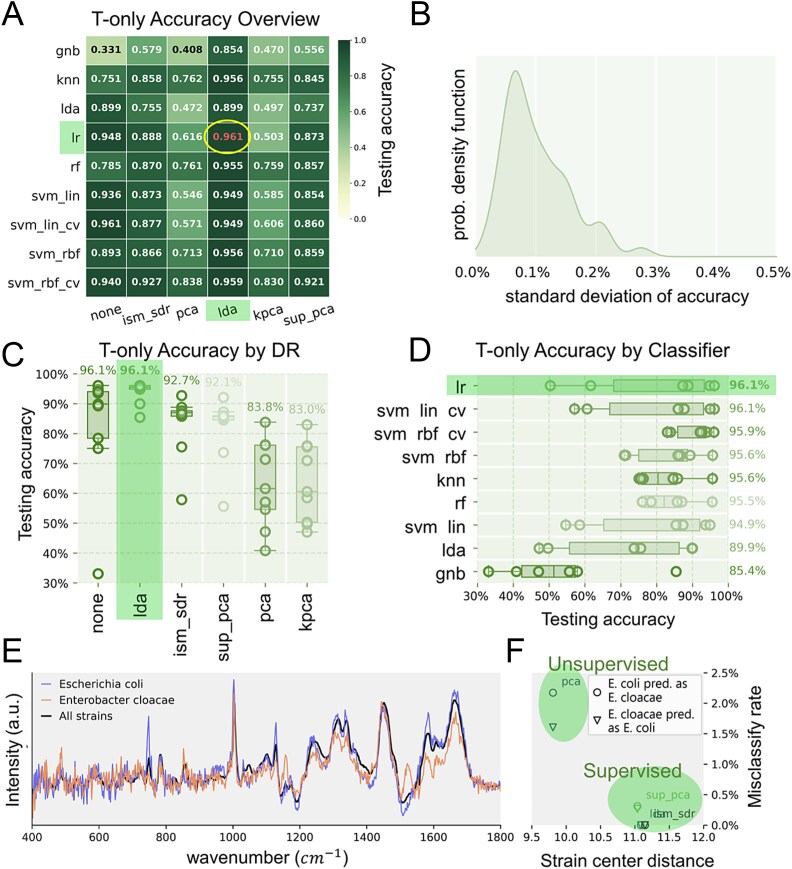
SCRS-based organism taxonomy identification without accounting for cell growth stage (T-only). (**A**) 54 models were tested individually via 10-fold cross-validation (CV), using a dataset containing 17 208 cells of 36 strains. All dimension reduction (DR) methods were set with an output dimensionality of 35 based on independent tests (in Supplementary Fig. S2). The highest accuracy achieved was 96.1%. (**B**) Classification accuracies were marginally impacted by the randomness introduced by the partitioning of CV splits in comparison to the choice DR and classifier. The maximum standard deviation introduced by this randomness is ~0.3% (absolute value). (**C**) Classifier performances (as circles) under different DR (in columns), the values on top correspond to the top accuracy observed with each DR. (**D**) DR performances (circles) under different classifiers (rows), the values to the right correspond to the top accuracy per classifier. (**E**) The pre-DR average spectra of strains *E. coli* and *E. cloacae*. (**F**) Supervised DR resulted in larger post-DR distances than unsupervised DRs between the two strains shown in (e), benefiting lower misclassification rates (LR as the classifier).

A total of 54 models that incorporate combinations of six DR methods and nine classifiers were tested to classify the taxonomy labels of all acquired SCRS samples without accounting for the growth stage information (Supplementary Fig. S1), referred to as the T-only approach. Promising strain-level classification accuracies were achieved, with the best observation being achieved by LDA as a supervised DR and LR as a classifier (LDA + LR) at 96.1% accuracy (Fig. 1A). The accuracy was higher than the studies using a large (>10 000) dataset, typically 75%–95% [9, 24]. All DRs were set with an output dimensionality of 35 based on an independent test (Supplementary Fig. S2). In addition, the impact on accuracy from randomness in CV splits was also evaluated as insignificant (standard deviation <0.3%, Fig. 1B) in comparison to the model choices with larger magnitudes of impacts.

#### Supervised DR outperformed unsupervised DR

Figure 1C compares the performance of the nine classifiers when combined with different DR. BEST [45] concluded that all nine classifiers showed statistically significant improvements in accuracy when paired with a supervised DR (LDA, ISM_SDR, and SUP_PCA) in comparison to unsupervised DR (PCA and KPCA), despite the dominant usage of PCA [48]. One possible reason is that supervised DR could be more sensitive in capturing the taxonomy-relevant information in our SCRS dataset as shown in a case study investigating the strains of *Escherichia coli* and *Enterobacter cloacae* (Figs. 1E and F). These two strains were selected because they had the highest misclassifications between them, whereas occurring rarely to be misclassified as other strains. The supervised DRs (LDA, ISM_SDR, and SUP_PCA) resulted in a larger post-DR distance between the two strains than PCA. This indicates that supervised DR can better distinguish the differences between closely-related SCRS spectra, benefiting a lower misclassification rate (KPCA with 0.2 distance and ~1.5% error rate not shown in Fig. 1F). In addition, using no DR (labeled as “NONE”) achieved the same level of accuracy (96.1%) as LDA when paired with an appropriate classifier, but also exhibited the worst-case accuracy among all DRs (Figs. 1A and C). This implies that DR can potentially be used as a noise removal strategy to improve classifier performance. When no DR is employed, the classifier selection (including hyperparameter optimization) could become critical.

#### LDA + SVM outperformed the DR + classifier combination

Figure 1D shows when paired with the best candidate DR (LDA), linear classifiers like LR and SVM_LIN_CV outperformed more flexible nonlinear classifiers like SVM_RBF_CV. The outperformance was found statistically significant (BEST) despite the small margins. This suggests that linear classifiers are generally suitable for taxonomic classifications with an SCRS dataset similar to our size. The smaller variance of SVM_RBF/SVM_RBF_CV in comparison to LR and SVM_LIN suggests that their performances are less sensitive to the choice of DR. This indicates that traditional nonlinear classifiers can still outperform linear classifiers when DR failed to capture label-relevant information due to their higher flexibility.

### SCRS-based taxonomic benchmarking performance with cell growth stages (T/G)

To evaluate the taxonomic classification with cell growth stage prior knowledge, all 54 algorithm combinations were re-trained with the 17 208-sample dataset pre-separated into four smaller datasets each consists of a specific growth stage (Exp, S1, S2, and S3), referred to as the T/G method (Supplementary Fig. S1). This resulted in multiple models (algorithm combinations) each being trained with and predicting cells from specific growth stages separately, evaluated by 10-fold cross-validations as described previously. No cross-growth-stage predictions (e.g. using Exp dataset for training and S1 for testing) were made. Higher accuracies were observed with all tested models after incorporating the growth stage prior knowledge. The best accuracy observed was 99.2%, (3.1% higher than the previous results), achieving the high end of strain-level SCRS-based taxonomy identification performance in application publications. Interestingly, this trend was opposite to other findings that classifiers usually have accuracy gain with the increment of the training dataset size [49]. It is potentially because that the S1 phase exhibited the best consistency and distinguishability of taxonomy-related signals, which overcame the disadvantage of smaller training dataset size.

**Figure 2:**
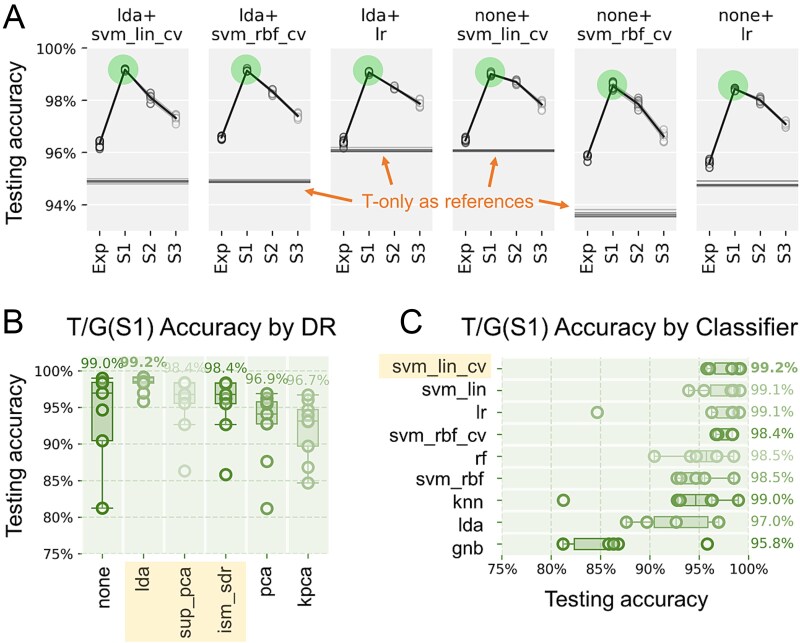
Comparison of taxonomy identification accuracy by models being trained by and predicting cells from specific growth stages (T/G). (**A**) Taxonomy identification accuracy varied depending on the cell growth stages, and the SCRS spectra obtained at stage S1 led to the highest prediction accuracy. A similar trend was observed in all six models previously identified as the algorithms in the best accuracy tier. Horizontal lines are T-only results from the respective algorithms for reference. Detailed accuracy distributions with the growth stage leading to the highest accuracy (S1) were shown in (b) and (c); other growth stages are included in Supplementary Fig. S3. (**B**) Classifier performances (as circles) under different DRs (in columns), the values on top correspond to the top accuracy observed with each DR. (**C**) DR performances (circles) under different classifiers (rows), the values to the right correspond to the top accuracy per classifier. *Note: Statistical significance exhibited between all growth stages in*  Supplementary Table S9.

49 out of the 54 models recognized S1 as the growth stage with the highest accuracy for taxonomy classification, including all six top-performing models identified previously (combination between {NONE, LDA} and {LR, SVM_LIN_CV, SVM_RBF_CV}) (Fig. 2A & Supplementary Fig. S3). The spectra collected from the S1 stage likely exhibited the best distinguishing features between taxonomies, suggesting that S1 is likely the optimal cell growth stage to sample for SCRS-based taxonomy identification. Future investigations are still required to elucidate its biochemical mechanisms and implications. One possible hypothesis is the change of metabolic state, particularly a transition from biomass reproduction to secondary metabolism at the beginning of the stationary phase [50]. Since SCRS encodes biomolecule signals as phenotypic fingerprints [51, 52], it hints at that the stationary phase could be intrinsically more closely related to species-differentiating biomolecules.

In addition, like the T-only results, supervised DRs were observed to outperform unsupervised DRs. LDA was identified as the outstanding choice in both the best-case accuracy and the smallest variance pairing with different classifiers (Fig. 2B). Figure 2C shows that linear classifiers, such as SVM_LIN and LR had a larger leading margin than the best non-linear classifier by 0.7–0.8% compared to Fig. 1D. MLP was also found not to necessarily outperform traditional classifiers in expense of training resources in both T-only and T/G approaches (Supplementary Text S4). This is a strong indication of linear separability between taxonomy labels when scoping cell SCRS from the same growth stage.

### Simultaneous growth stage and taxonomy identification (G-T)

We showed that growth stage prior knowledge could enhance the taxonomy classification accuracy in previous sections. To investigate the possibility of recovering growth stage information in the absence of prior knowledge and its potential impact on taxonomy identification, a 2-step growth-stage-then-taxonomy classifier ensemble (referred to as the G-T approach) was tested. This was achieved by adding a growth stage classification step before the taxonomy classification (Supplementary Fig. S1).

####  

**Figure 3:**
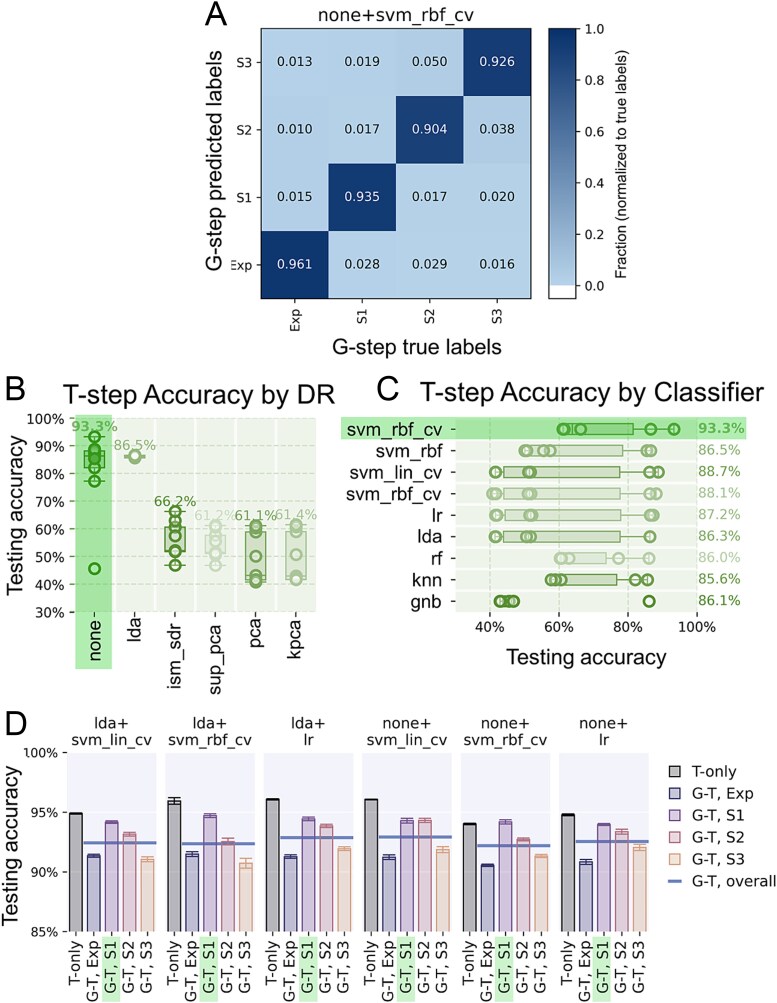
Taxonomy identification accuracy of the growth stage-taxonomy 2-step classification model (G-T model). (**A**) Confusion matrix of the growth stage classification, using NONE+SVM_RBF_CV identified as the model with the highest observed accuracy (93.3%). (**B and C**) Comparisons between DR and classifiers indicate that NONE+SVM_RBF_CV is the top candidate model for growth stage classification. (**D**) Comparison between G-T and T-only taxonomy identification accuracy.

***Growth stage classification (G-step).*** All 54 models were tested to select the one with the best accuracy in predicting the cell growth stages based on the SCRS spectrum of each cell, with an output dimensionality of 3 (the highest possible to run LDA with a 4-class dataset) of all DR methods. Figures 3B and C summarize the comparisons of resulting accuracy from 10 repeats of 10-fold CVs; accuracies of individual models are shown in Supplementary Fig. S4. NONE+SVM_RBF_CV was recognized as the best model as it achieved 93.3% average accuracies, superseding the second-ranked model by 4.6% (NONE+SVM_LIN_CV). Therefore, this model combination was selected for growth stage classification in later explorations. LDA was recognized as the best DR method by at least a 20.3% margin in best-case accuracies; though in this case skipping DR (NONE) was better by a margin of 6.8%. In addition, the superior performance of SVM_RBF_CV indicates that in the classification of cell growth stages, a nonlinear classifier and fine-tuning may benefit an optimized accuracy. The confusion matrix in Fig. 3A shows that the misclassification rate was higher between the three stationary stages (≥ 1.6%) than between the exponential and any stationary phase (≤ 1.5%), possibly due to a higher SCRS similarity within the three stationary stages, than between the exponential and stationary stages.

***Taxonomy identification (T-step).*** The T-step consists of four taxonomy predicting ***DR + classifier combinations*** each being trained by and predicting cell spectra of a specific growth stage predicted by the G-step. Their setup is similar to those in the T/G approach except for that the growth stage pre-separation is done the G-step rather than using the ground truth labelled as in the original dataset*.* Note that this G-T approach here attempts to recover the growth stage information by mimicking the fact that cellular growth stage is unknown in their living environment. Figure 3D shows the T-step accuracy with the G-step using the previously identified best performer (NONE+SVM_RBF_CV) for simplicity. In addition, the T-only results were included for references as the it is arguably the most common in related applications. Overall, the T-step achieved 91.1%–94.4% accuracy. Though they were lower than the T-only (94.9%–96.1%, see statistical significances in Supplementary Fig. S5), it provided a sufficiently high accuracy (> 91%) of taxonomy identification and growth stages classification simultaneously. This implies significance in practical biological and environmental applications, where SCRS data retrieved from real organism samples often represent varying and diverse growth stages and conditions, and thus it would be meaningful and relevant to have both taxonomic identification and growth stage information. We also observed that the S1 phase was still recognized as the optimal growth stage in general for SCRS-based taxonomy identification, being consistent with the previous discussion. On the other hand, the accuracy drop observed in G-T in comparison to T-only could be caused by two reasons: (i) errors of the growth stage classification might have propagated to the taxonomy classification; (ii) growth stage and taxonomy classification may inference with each other due to potential overlap of key features. A larger training dataset from comprehensive cell phylogeny diversity and growth stages might help reduce this inference. For example, we noticed that the key features of cell growth stage and cell phylogeny may overlap, and thus, future datasets labelled with both taxonomy and growth stage labels therefore help to improve the ML model to reliably analysis this overlapping and ultimately benefit accuracy improvements in robust simultaneous growth stage and taxonomy identification.

### Extending SCRS-based taxonomic identification models from pure cultures to real environmental microbiomes

**Figure 4:**
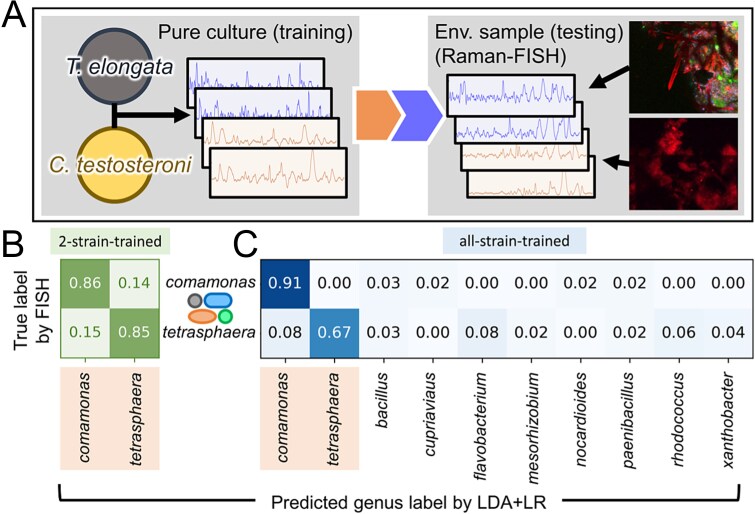
Accuracy assessment of SCRS-based taxonomic identification of *Comamonas* and *Tetrasphaera* spectra from an environmental sample at a wastewater treatment plant. (**A**) The training dataset contains only pure culture SCRS, and the testing dataset is labelled with FISH-acquired taxonomy (*Comamonas* and *Tetrasphaera* genera). (**B and C**) indicate two tested cases, including (**B**) trained using only *Comamonas* and *Tetrasphaera* pure culture spectra; (**C**) trained using *Comamonas*, *Tetrasphaera*, and all 36 strains from the previous dataset; the merged dataset was re-balanced at genus level. The predicted genus labels without any predictions were not shown.

SCRS-based taxonomic identification requires the establishment of SCRS library of relevant organisms with known taxonomy labels either via pure culture collections or SCRS retrieval of from the environmental samples using techniques such as Raman-FISH. Whether the model can overcome all the unavoidable environmental and instrumental noises and variations among the training SCRS library (classification labels) and SCRS data obtained from environmental samples holds the key to its applications. Hence, a case study was conducted to assess whether SCRS acquired from pure culture could be effectively used as references for taxonomic classification of SCRS spectra obtained from real environmental samples (Fig. 4). Two major genera, *Tetrasphaera* and *Comamonas*, were selected because of their keystone roles in wastewater phosphorus removal [53]. The LDA + LR model (the optimal in above section) was tested in two independent cases both using setups similar to T-only. Firstly, the training dataset with only *Tetrasphaera elongata* and *Comamonas testosteroni* pure cultures yielded an 85% genus-level accuracy in SCRS-based identification, indicating that predicting environmental SCRS spectra using references from pure culture is feasible (Fig. 4B). Secondly, the existing SCRS reference database of 36 strains was extended to 36 strains by adding the only *T. elongata* and *C. testosteroni* pure cultures, then the LDA + LR model was trained for the extended database. Figure 4C shows the confusion matrix predicting the same testing dataset as Fig. 4B, with an overall accuracy of 79%. The lower accuracy with the real environmental samples compared with the previous accuracy for pure cultures (96.1%) accuracy was likely attributed to the Raman-FISH process that impact the SCRS signal due to cellular structure alternations and background fluorescence in the FISH step [54]. It is worth noting that in real applications FISH will not be required, therefore the accuracy is expected to be higher than in this case where we employed Raman-FISH for validation.

## Discussion

### SCRS classification calls for standardized model selection and optimization

In SCRS-based taxonomy identification, this study showed that standardized model selection and optimization are crucial. The choice of model can introduce accuracy variations significantly larger than those caused by intrinsic cell factors such as growth stages, with differences up to 41.4% observed between models (e.g. LDA + SVM_LIN_CV vs. NONE+GNB). A similar magnitude of variances were also observed in other studies with less comprehensive comparisons [9, 55]. We confirmed that optimized algorithm choice could potentially achieve an accuracy higher than the typical range reported by recent studies using similarly-sized datasets [9, 24]. Traditional classifiers (e.g. LR and SVM_LIN) may better suffice taxonomy identification tasks on datasets sized similar to ours than NN, because of a less potential of overfitting and faster training. This may apply to SCRS datasets commonly seen in studies typically ranging from several hundred to 10 000. However, further investigations are expected necessary when SCRS acquisition [55] becomes more throughput to enable larger datasets.

Applying DR can enhance the signal-to-noise ratio, reduce irrelevant information, and mitigate overfitting in high-dimensional SCRS data. DR also offers other practical benefits like reduced dataset size, memory usage, and faster training [56]. Optimizing DR output dimensionality is recommended in real application when DR is employed, e.g. but not limit to brute-forcing through a viable dimensionality range (like our optimization as shown in Supplementary Fig. S2).

### Impact of cell growth variances on SCRS-based taxonomy identification

This study is the first to comprehensively compare various DR and classification methods for SCRS-based taxonomy identification, evaluating the impact of cell growth stages on accuracy. LDA + LR and LDA + SVM_LIN_CV performed best, with minor accuracy differences (0.3%–2.8%) across different approaches, achieving 96.1% accuracy even without prior knowledge of growth stage. This is crucial for biomedical and environmental applications where pre-identifying cell growth stages is challenging. On the other hand, with regard to application scenarios where identifying cellular growth status is critical (such as cancer traits or growth-stage-sensitive metabolic states for environmental microbes), correctly identifying the cell growth stage can benefit more accurate cell taxonomy identification according to the statistical significance we observed.

Our results indicate that cells at the early stationary phase are optimal for SCRS classification, as strain-specific metabolisms are highly active, enhancing taxonomy identification accuracy [57, 58]. In contrast, the exponential phase focuses on general metabolic activities, and late stationary phases lead to biomass decay, reducing accuracy [59]. Therefore, we suggest that SCRS spectra reference libraries should include growth stage labels for improved accuracy [59], as biasedly using spectra from a particular growth stage as classification references may lead to a drastic deterioration of the accuracy in predicting cell taxonomy from other growth stages (Supplementary Fig. S6).

In this study, we introduced the G-T approach, a multi-label classification that predicts cell growth stage and taxonomy simultaneously, achieving 93.3% and 92.8% accuracy, respectively. This approach allows for growth-stage preselection before taxonomy identification, using full spectra and non-linear classifiers, and can also filter out high-noise or burnt biomass spectra, enabling a more flexible and more seamless integration of multi-dataset analysis.

### ML-enabled SCRS revealed phenotype–genotype linkage for environmental applications

Establishing phenotype-genotype linkages through simultaneous taxonomic identification and metabolic profiling is challenging, especially in environmental and ecological studies. SCRS has emerged to be a promising tool for non-invasive, cultivation-free platform for phenotyping, however, SCRS-based taxonomy identification is still nascent. Previous models lacked benchmarking and often ignored key biological factors like cellular growth status, with no testing on real environmental samples. Our study is the first to demonstrate that ML-enabled SCRS can identify both phenotypes (growth stages) and genotypes (taxonomy) with 85% genus-level accuracy, promising that SCRS-based taxonomy identification is feasible for environmental applications (Fig. 4B). As the SCRS reference library grows and we better interpret SCRS spectra for cellular biochemical composition, the data will reveal new insights into phenotypic and metabolic traits (Supplementary Text S5  **and**  Fig. S10). We expect that detailed SCRS feature analyses can contribute to more accurate metabolic traits and taxonomy identification for future applications.

To our best knowledge, this is the first endeavor at SCRS-based taxonomic identification using real environmental samples. We demonstrated that lab-cultivated pure cultures can serve as references for classifying environmental cells, despite that accuracy can be affected by environmental variables and FISH processing. Accuracy is expected to be improved by eliminating the FISH that could introduce noises as explained previously. In the future, the database can be expanded in all three dimensions: more comprehensive species/strain labels, deeper sampling depth per label, and more dual-labelled datasets with both taxonomy and growth stage labels to further improve the SCRS-based taxonomic identification.

## Conclusion

This study advances the development of ML-enabled SCRS technologies as robust tools for linking high-resolution phenotypes and genotypes at the single-cell level, promising broad environmental applications. This technology can enable high-throughput screening of functional organisms, monitor microbial dynamics in response to environmental changes, and unravel complex microbial interactions *in situ*. By enhancing our ability to identify and categorize microbial entities based on their genetic and phenotypic characteristics, it also allows for a detailed understanding of their functional roles within ecosystems. In environmental conservation, it can identify key microbes involved in biodegradation, nutrient cycling, and carbon sequestration. In agriculture, it aids in discovering and monitoring beneficial microbes that promote soil health and plant growth, leading to more sustainable practices. The broad applicability of this technique paves the way for a deeper and functionally oriented understanding of microbial communities across various ecological studies.

## Supplementary Material

si_ycaf015

## Data Availability

The original SCRS dataset can be accessed per authors’ request. All directly related data are within the manuscript and its Supplementary Information files.

## Code availability

The code can be accessed in GitHub Repository: https://github.com/aprilgugroup/SCRS-clf-ensemble
