# Supplementary material for: Towards high-accuracy bacterial taxonomy identification using phenotypic single-cell Raman spectroscopy data
Source: ISME Commun. 2025 Mar 9;5(1):ycaf015. doi: 10.1093/ismeco/ycaf015 (PMC11910137; doi:10.1093/ismeco/ycaf015)
Supplement: si_ycaf015 [file si_ycaf015.pdf]

SUPPLEMENTARY INFORMATION

Towards High-Accuracy Bacterial Taxonomy Identification Using Phenotypic Single-Cell Raman Spectroscopy (SCRS) Data

Guangyu Li [1, †], Zijian Wang [2, 3, †], Chieh Wu [4], Dongqi Wang [5], David R. Kaeli [4], Jennifer G. Dy [4], Kilian Q. Weinberger [6], and April Z. Gu [1, 3, *]

[1] School of Civil and Environmental Engineering, Cornell University, Ithaca, NY, United States
[2] Department of Biological and Environmental Engineering, College of Agriculture and Life Sciences, Cornell University, NY, United States
[3] Center for Research on Programmable Plant Systems, 103 Rice Hall, Cornell University, Ithaca, NY, United States
[4] Department of Electrical and Computer Engineering, Northeastern University, Boston, MA, United States
[5] Department of Municipal and Environmental Engineering, School of Water Resources and Hydro-Electric Engineering, Xi’an University of Technology, Xi’an, Shaanxi, China
[6] Department of Computer Science, Cornell University, Ithaca, NY, United States
[†] These authors contributed equally to this study
[*] Corresponding author: aprilgu@cornell.edu

TERMS & ABBREVIATIONS

BEST: Bayesian estimation supersedes the t test [1] (an alternative to the t-test in two-sample hypothesis testing).

CV: cross-validation.

DR: dimensionality reduction.

FDR: false discovery rate.

MCMC: Markov Chain Monte Carlo.

RBF kernel: radial basis function kernel (Gaussian kernel as an alternative name).

Cell Growth Stages in the Dataset:

Exp (microbial cell growth stage): exponential phase.

S1 (microbial cell growth stage): the beginning of stationary phase.

S2 (microbial cell growth stage): the midpoint of stationary phase.

S3 (microbial cell growth stage): the end of stationary phase.

Dimensionality Reduction Methods:

None: no dimensionality reduction is used.

ISM_SDR: iterative spectrum method, developed by Wu et. al. [2]

PCA: principal component analysis [3].

LDA: linear discriminant analysis [3], can be used both as dimensionality reduction and classifier.

KPCA: kernel PCA (with RBF kernel) [4].

SUP_PCA: supervised PCA [5].

Classifiers:

GNB: Gaussian naïve Bayes classifier [3].

KNN: k-nearest neighbor classifier [6].

LDA: linear discriminant analysis [3], can be used both as dimensionality reduction and classifier.

LR: logistic regression [6].

RF: random forest (ensemble of 100 decision trees) [3].

SVM: support vector machine; C-SVM is used in this study.

SVM_LIN: C-SVM with linear kernel [7].

SVM_LIN_CV: C-SVM with linear kernel, parameter C optimized via CV.

SVM_RBF: C-SVM with RBF kernel [3].

SVM_RBF_CV: C-SVM with RBF kernel, parameter C, and RBF kernel scaling factor σ optimized via CV.

FIGURES

The workflow overview of this study was represented in **Fig. S1**.

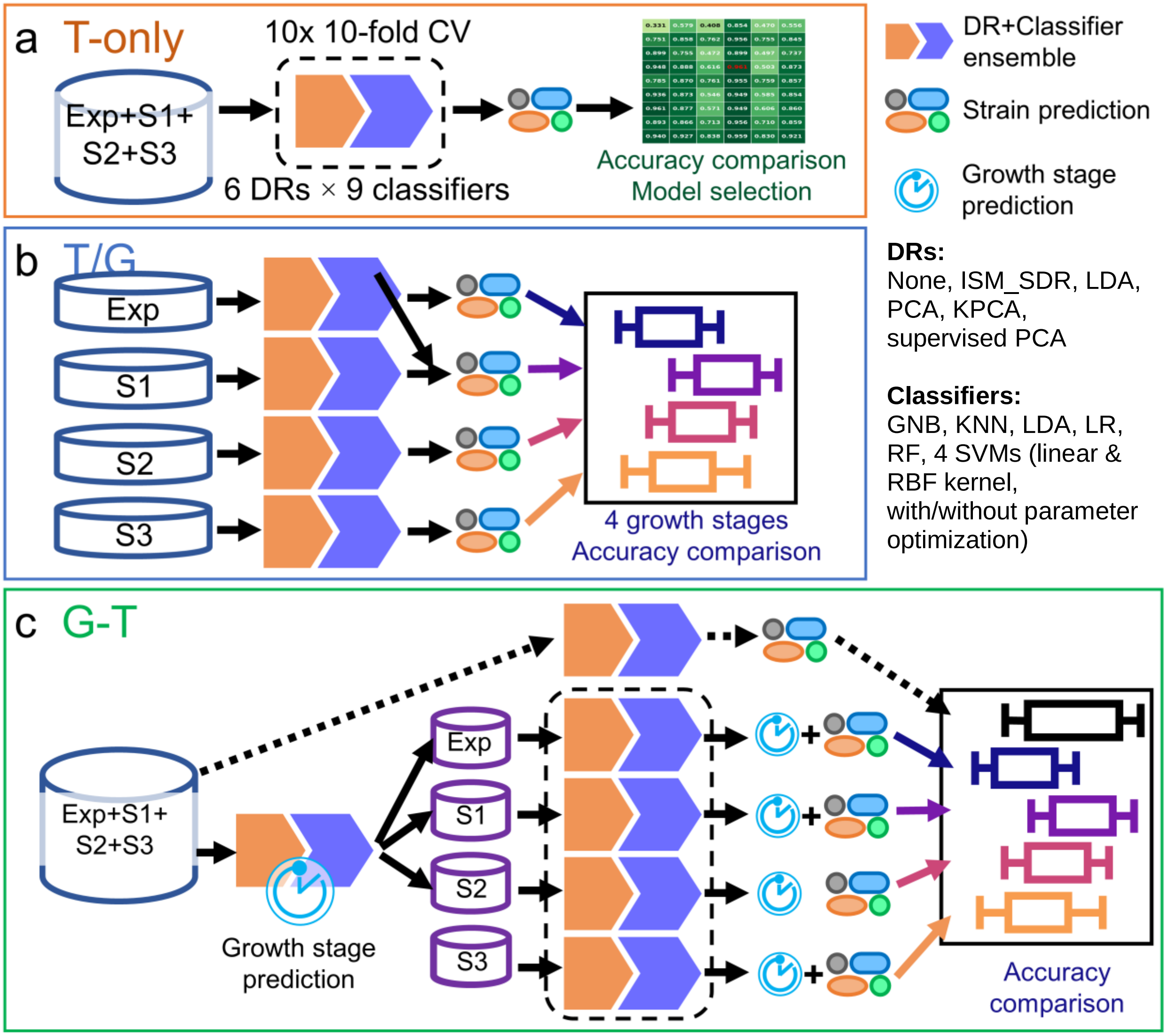

Fig. S1: Workflow of taxonomy identification, using an SCRS dataset containing 17,208 spectra from 36 strains during 4 different growth stages (Exp: exponential stage; S1-S3: beginning, midpoint, and end of the stationary stage; stages are determined by growth curve). **(a)** A total of 54 models (6 DRs × 9 Classifiers) were compared for their strain-level taxonomy prediction accuracy evaluated by 10-fold CV using all the spectra for 36 strains without accounting for growth stage prior knowledge (T-only approach). **(b)** 54 models were benchmarked with variants each being trained and predicting a specific growth stage ground truth (T/G approach). **(c)** A 2-step growth-stage-then-taxonomy (G-T) classifier ensemble was tested to recover growth stage information when it is not available as prior knowledge. The taxonomy prediction results were compared with previous results.

Output dimensionality optimization results were shown in **Fig. S2**, with each DR and classifier combination tested under a variety of output dimensionality from 2 to 35. No dimensionality beyond 35 was tested as it is the highest value possible to run LDA on a 36-class dataset. The optimal dimensionality 35 based on the observations.

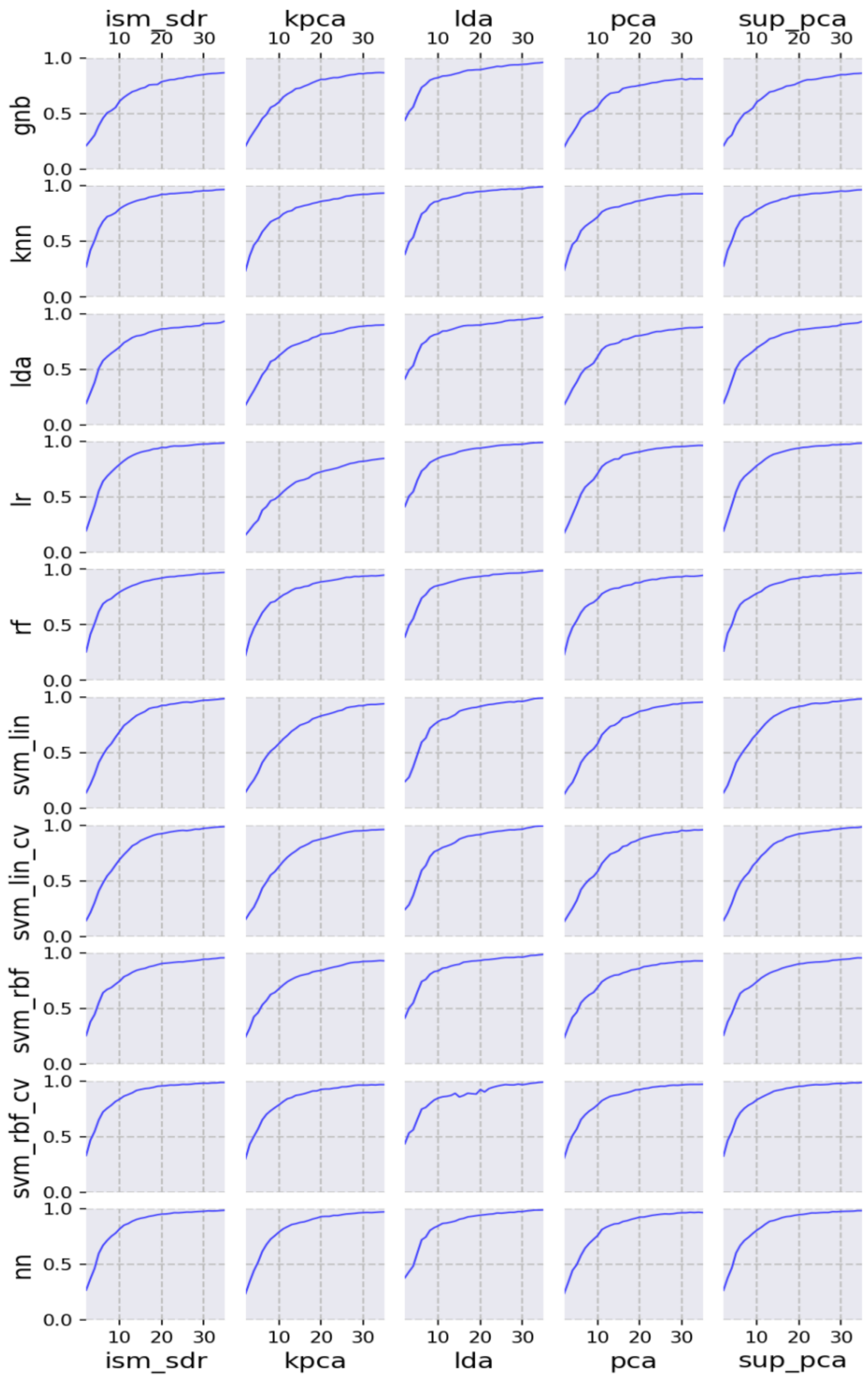

Fig. S2: Accuracy versus output dimensionalities. Each plot shows the result of a tested model built from the corresponding dimensionality reduction method (in columns) and the classifier (in rows). 35 output dimensions is

chosen for its best performance tested across all models and being the highest number possible with LDA (with a 36-class training dataset).

Fig. S3 shows the comprehensive DR and classifier comparisons in the T/G approach, in complementary to **Main text Figs. 2b&c**.

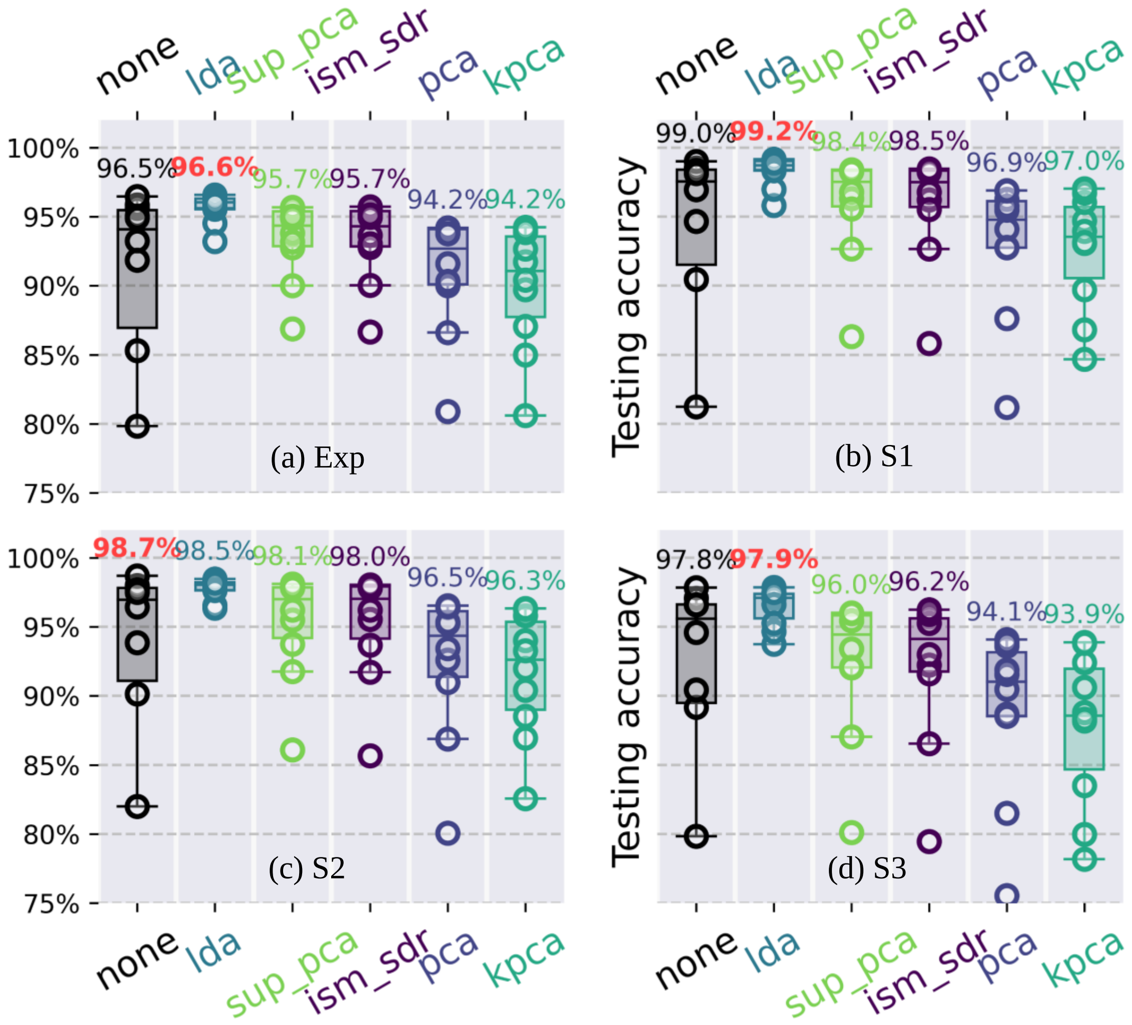

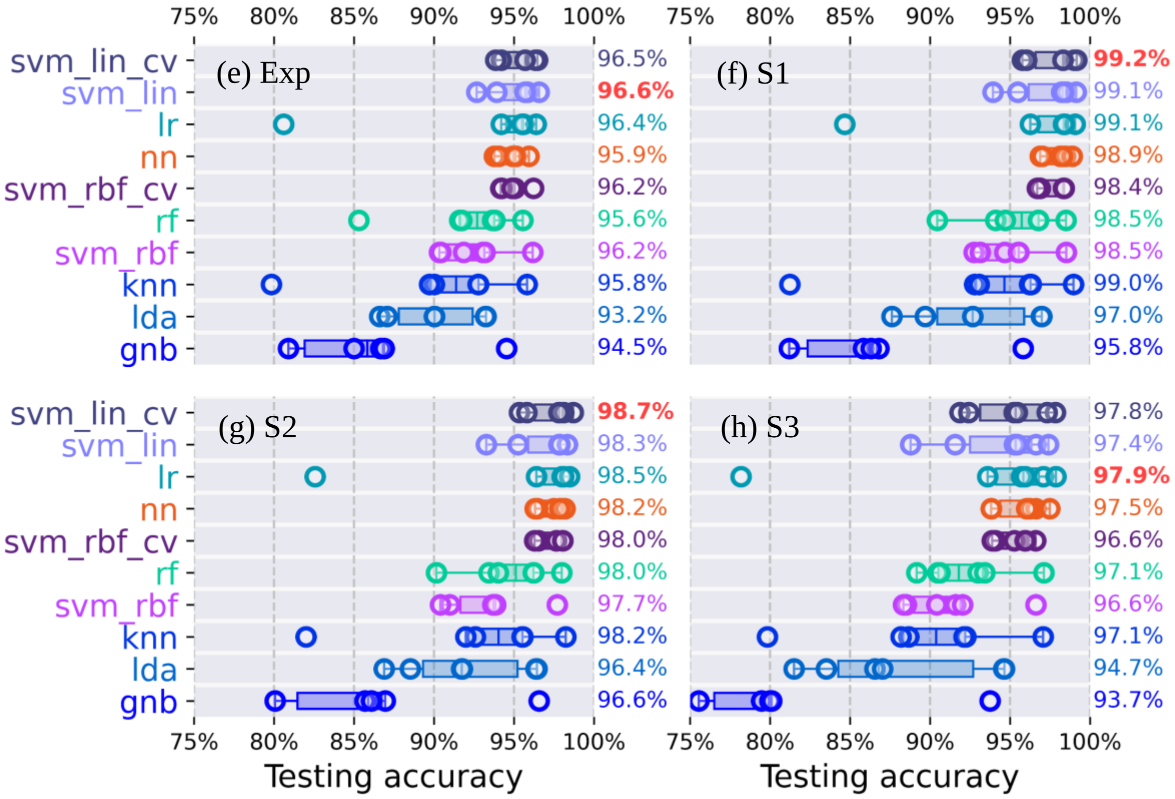

Fig. S3: (a-d) Classifier performances (as circles) under different DRs (in columns), the values on top correspond to the top accuracy observed with each DR. **(e-h)** DR performances (circles) under different classifiers (rows), the values to the right correspond to the top accuracy per classifier. All models are trained and tested in the T/G approach.

Fig. S4 shows the accuracies using different DRs (in columns) and classifiers (in rows) in growth stage identification (G-step) of the growth-stage-then-taxonomy (G-T) approach.

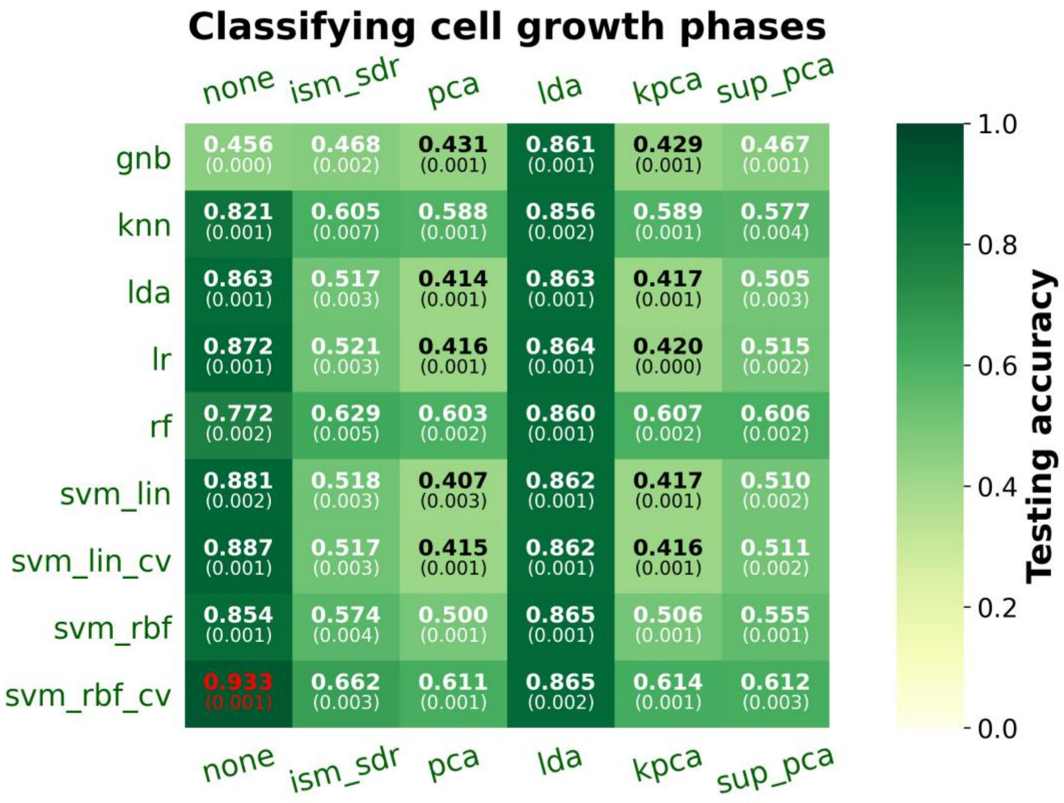

Fig. S4: Average accuracies of cell growth stage prediction by 54 models (combinations by 6 dimensionality reduction (in columns) and 9 classifiers (in rows)). The larger, bolded values are average accuracies observed from 10 individual runs of 10-fold cross-validation of the respective model; the smaller values in parenthesis are the standard deviations respectively. The best-case accuracy is highlighted in red.

Fig. S5 shows the accuracy comparison between the T-only and the taxonomy identification (T-step) of the G-T approach. The accuracies are identical to **Main text Fig. 4d** with extra statistical significance results showing as binary grids. A solid cell means the respective accuracy difference has statistical significance, and a hollow cell means insignificance. In addition, S1 was recognized

as the growth stage that has the highest classification accuracy despite potential errors propagated from the first-step classification.

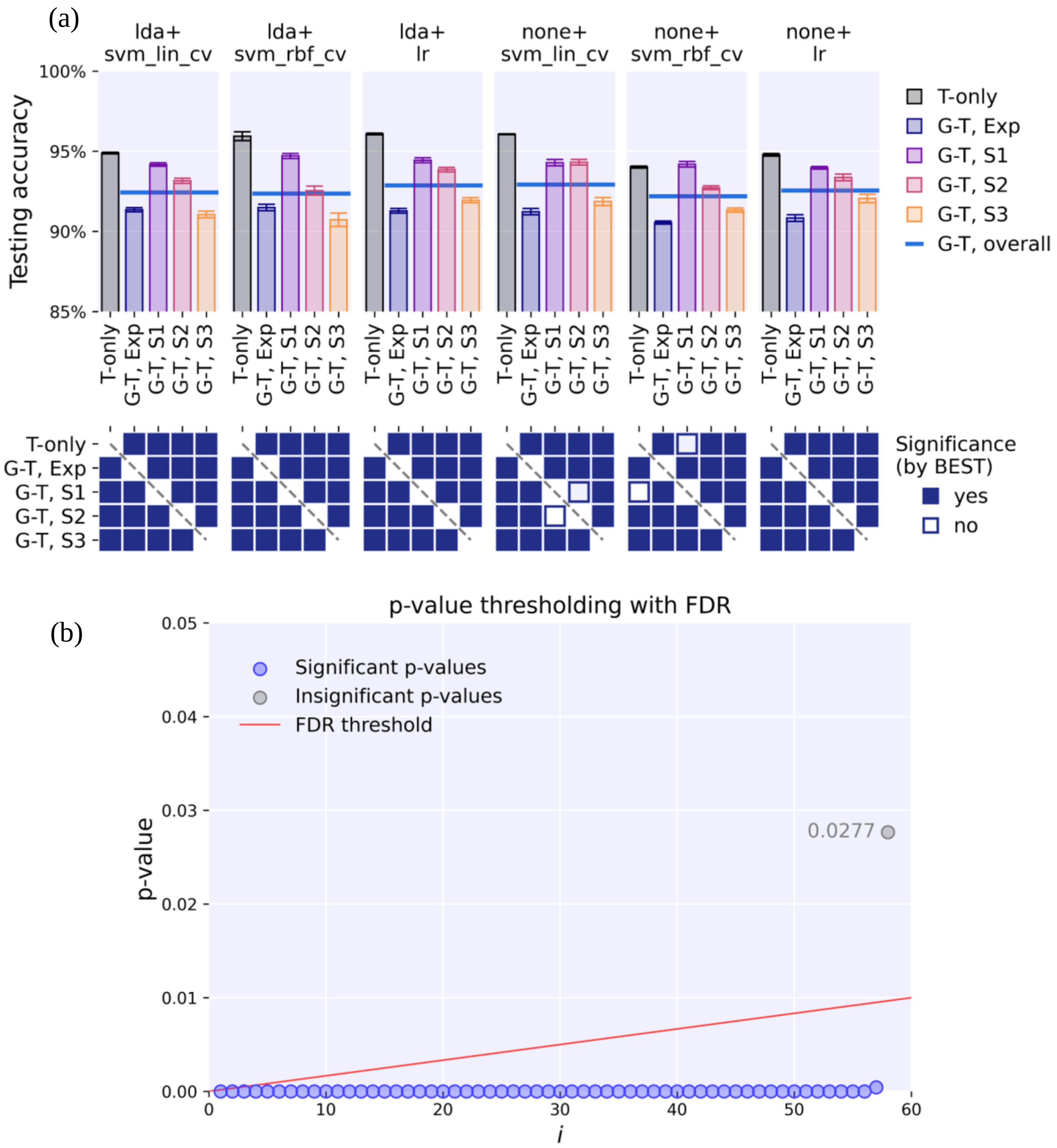

Fig. S5: (a) Comparison of T-step taxonomy identification accuracy of the G-T approach, with T-only results included as references. The Boolean matrices below show if the accuracy differences using different models are statistically significant, as being assessed by BEST [1] with α=0.01 and FDR thresholding to control false positives. **(b)** Plot for the FDR thresholding, with 58 p-values shown and 2 higher values (being insignificant) off the plot. The p-values below the red threshold line are considered significant.

Fig. S6 shows dramatic deterioration of cross-growth-stage prediction accuracy. The setup is similar to the T/G approach except that the training and predicting cells are from different growth stages. For example, predicting S3 cell spectra with models trained by Exp cell spectra.

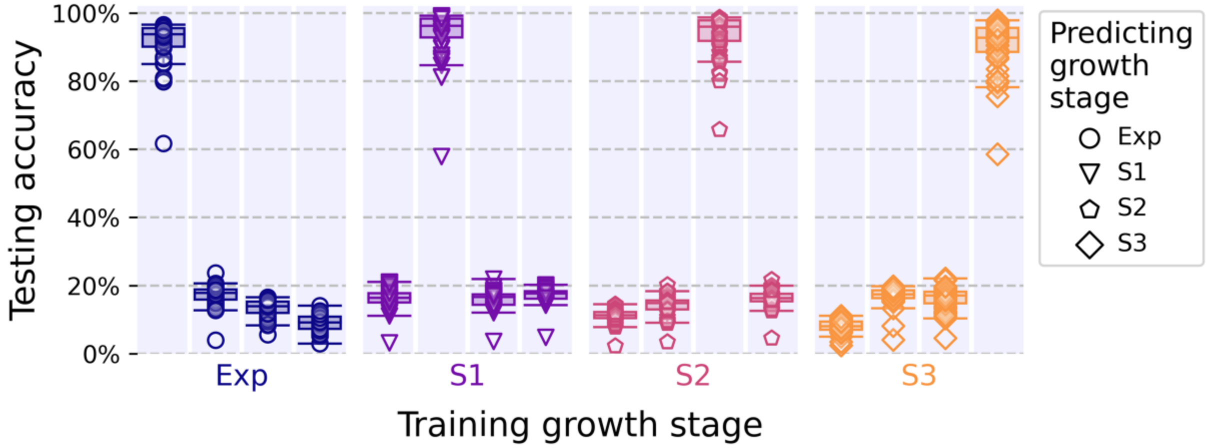

Fig. S6: Accuracy comparison between cross-growth-stage taxonomy prediction (training and testing spectra are for different growth stage cells) and ordinary cross-validation (training and testing spectra are from the same growth stage cells). All cross-growth-stage predictions had dramatically deteriorated accuracies.

Supplementary Table S1

Table S1: The list of 36 microorganism strains used in this study.

ID	NRRL No.	Name	Source	Functions and Traits	Medium Number	Condition
**1**	HD-511	*Bacillus thuringiensis*	Natural-occurring, soil-borne strain	natural insect control	1	28 ℃
**2**	B-23289	*Enterobacter cloacae*	Human	biological control of plant diseases	1	28 ℃
**3**	B-371	*Paenibacillus polymyxa*	soil, plant tissues and marine sediments	biofertilizer and biocontrol agent	1	28 ℃
**4**	B-14869	*Pseudomonas chlororaphis*	soil	soil inoculant and biocontrol agent	1	28 ℃
**5**	B-802	*Delftia acidovorans*	soil	plant growth promoting	1	28 ℃
**6**	B-14938	*Pseudomonas putida*	soil	bioremediation	15	28 ℃
**7**	B-41093	*Bacillus species*	/	/	1	30 ℃
**8**	B-14472	*Bacillus subtilis subsp. Spizizenii*	soil and the gastrointestinal tract of ruminants and humans	symbiosis, lower the insoluble phosphorus in soil	1	28 ℃

**9**	B-14368	*Bacillus licheniformis*	soil	plant growth promoting	1	28 ℃
**10**	NRS-269	*Bacillus megaterium*	common in soil, but also in food and surfaces	biocontrol of plant diseases, nitrogen fixation	1	28 ℃
**11**	B-3159	*Arthrobacter ramosus*	beech forest soil	bioremediation	1	28 ℃
**12**	B-23299	*Paenibacillus denditriformis subsp. Dendron*	soil, water, rhizosphere, vegetable matter, forage and insect larvae	antimicrobial-producer	1	28 ℃
**13**	B-775	*Pseudomonas stutzeri*	soil	nitrogen fixation	1	28 ℃
**14**	B-59347	*Stenotrophomonas rhizophila*	stems, leaves, or the rhizosphere	symbiosis, plant growth promoting	1	30 ℃
**15**	B-787	*Lactobacillus plantarum*	fermented food, anaerobic plant	biocontrol	12	28 ℃
**16**	B-59838	*Escherichia coli serotype O157:H7*	lower intestine, WWTPs	cause disease	15	37 ℃
**17**	B-41609	*Mesorhizobium amorphae*	soil	root nodule bacteria, plant growth promoting	1	28 ℃

**18**	B-14838	*Xanthobacter flavus*	soil	bioremediation	1	28 ℃
**19**	B-14393	*Bacillus amyloliquefaciens*	soil	fight root pathogen, plant growth promoting	1	28 ℃
**20**	BD-147	*Bacillus pumilus*	commonly found in soil	agricultural fungicides	1	28 ℃
**21**	B-16357	*Streptomyces atrovirens*	soil	produces plant hormone: indole-3-acetic acid	20	28 ℃
**22**	B-1574	*Rhodococcus erythropolis*	soil, water	bioconversion and bioremediation	6	28 ℃
**23**	B-14799	*Bacillus mycoides*	soil	biocontrol	1	28 ℃
**24**	B-14811	*Bacillus mycoides Flugge 1886*	soil	biocontrol	1	28 ℃
**25**	B-348	*Bacillus mycoides*	soil	biocontrol	1	28 ℃
**26**	NRS-273	*Bacillus mycoides Flugge 1886*	soil	biocontrol	1	28 ℃
**27**	B-617	*Bacillus pseudomycoides*	soil	similar phenotypic characteristics to B. mycoides but	1	28 ℃

				distinct genomic information		
**28**	B-14733	*Flavobacterium johnsonae*	commensal bacteria in soil and water	opportunistic pathogens	1	28 ℃
**29**	B-14842	*Flavobacterium aquatile*	water	glucose fermenter	1	28 ℃
**30**	B-2648	*Flavobacterium resinovorum*	soil	unknown	1	30 ℃
**31**	B-59409	*Enterobacter soli*	soil	lignin-degrading	1	28 ℃
**32**	B-14690	*Cupriavidus necator*	soil	fix carbon dioxide as a carbon source, use the urea in urine as a nitrogen source, and use hydrogen as an energy source to create dense cultures that could be used as a source of protein	1	28 ℃
**33**	B-3159	*Arthrobacter ramosus*	beech forest soil	bioremediation	1	28 ℃

**34**	B-3157	*Nocardioides simplex*	soil, water	bioconversion and bioremediation	1	28 ℃
**35**	B-3704	*Escherichia coli*	lower intestine, WWTPs	cause disease	1	28 ℃
**36**	B-23264	*Enterobacter cloacae*	Human	biological control of plant diseases	1	28 ℃

Note: Detailed bacterial culture procedures were described here. (1) break the glass tube wrapped in paper towel, transfer the pellet into 1 mL of 30% glycerol and hand-vortex it, (2) store several replicates in -80℃ fridge, (3) cultivate the inoculum in a solid medium plate and then transfer a single microcolony into the liquid medium, and (4) measure the growth curve and prepare the samples of bacteria in different growth phase for Raman spectroscopy as described previously1. The USDA-ARS Culture Collection is the provider of the 36 strains [9].

Text S1: Experimental Information of the 36-strain SCRS Dataset Preparation

Bacterial culture condition and sampling time point. The detailed information about the medium and culture conditions for all the strains were provided in **Table S2-S6**. OD was measured at a wavelength of 600nm in a Microplate Reader (Synergy H1 Multi-Mode, Biotech, VT, USA). In order to capture the metabolic phenotype dynamics under various growth stages, we collected >100 SCRS spectra at 4 sampling time points. This is considered sufficient for pure cultures in leu of the statistical analysis of minimal numbers of cells as detailed by Li et. al. [10]. The time points to sample cells for SCRS acquisition were determined individually for each strain by monitoring the optical density using 600nm wavelength light (OD_{600}). In total, one time point and 3 time points were sampled from the exponential phase and the stationary phase respectively. The exponential time point samples were taken when the OD_{600} value was around half of its maximum (denoted as Exp). The beginning-of-stationary time point samples were taken when the OD_{600} value first reached its maximum (denoted as S1). The end-of-stationary time point samples were taken when the OD_{600} value started to decrease (denoted as S3). The mid-point-of-stationary time point samples were taken at the midpoint of S1 and S3 (denoted as S2). The detailed OD_{600} value was listed in **Table S7**.

Table S2: Medium No. 1 (source: NRRL database for prokaryote)

**Tryptone-Yeast Extract-Glucose Agar (TGY)**	
Tryptone	5 g/L
Yeast extract	5 g/L
K_2HPO_4	1 g/L
Glucose	1 g/L
Agar	15 g/L

Table S3: Medium No. 6 (source: NRRL database for prokaryote)

**Yeast Extract-Malt Extract-Peptone-Glucose Agar (YM for yeasts)**

Yeast extract	3 g/L
Malt extract	3 g/L
Peptone	5 g/L
Glucose	10 g/L
Agar	15 g/L

Table S4: Medium No. 12 (source: NRRL database for prokaryote)

**Liver Infusion Broth (LIB)**	
Dehydrated, pre-pared Liver Infusion Broth	35 g/L

Table S5: Medium No. 15 (source: NRRL database for prokaryote)

**Trypticase Soy Agar (TSA)**	
Dehydrated, pre-prepared BBL Trypticase Soy Broth	30 g/L
Agar	15 g/L

Table S6: Medium No. 20 (source: NRRL database for prokaryote)

**N-Z Amine with Soluble Starch and Glucose Agar (NSG)**	
Glucose	10 g/L
Soluble Starch	20 g/L
Yeast extract	5 g/L
N-Z Amine type A	5 g/L
CaCO3	1 g/L
Agar	15 g/L

 Table S7: Growth Stage of 36 Strains

ID	NRRL No.	Name	OD600				Time (hour)			
			mid-log	S1	S2	S3	mid-log	S1	S2	S3
**1**	HD-511	*Bacillus thuringiensis*	0.5-0.6	1.1-1.2	1.1-1.2	1.1-1.2	7	12	20	28
**2**	B-23289	*Enterobacter cloacae*	0.5-0.6	1.1-1.2	1.1-1.2	1.1-1.2	6	12	19	26
**3**	B-371	*Paenibacillus polymyxa*	0.5-0.6	1.2-1.3	1.2-1.3	1.2-1.3	32	52	116	180
**4**	B-14869	*Pseudomonas chlororaphis*	0.6-0.7	1.0-1.1	1.0-1.1	1.0-1.1	6	9	32.5	56
**5**	B-802	*Delftia acidovorans*	1.0-1.2	2.1-2.2	2.1-2.2	2.1-2.2	48	72	117	162
**6**	B-14938	*Pseudomonas putida*	0.6-0.7	1.2-1.3	1.2-1.3	1.2-1.3	16	24	43	62
**7**	B-41093	*Bacillus species*	0.5-0.6	1.1-1.2	1.1-1.2	1.1-1.2	8	13	21.5	30
**8**	B-14472	*Bacillus subtilis subsp. Spizizenii*	1.0-1.1	2.0-2.2	2.0-2.2	2.0-2.2	4	9	19.5	30
**9**	B-14368	*Bacillus licheniformis*	1.0-.12	2.0-2.3	2.0-2.3	2.0-2.3	11	18	34.5	51
**10**	NRS-269	*Bacillus megaterium*	0.6-0.7	1.1-1.2	1.1-1.2	1.1-1.2	22	48	114	180
**11**	B-3159	*Arthrobacter ramosus*	0.6-0.8	1.1-1.2	1.1-1.2	1.1-1.2	14	38	113	188
**12**	B-23299	*Paenibacillus denditriformis subsp. Dendron*	0.5-0.6	1.0-1.2	1.0-1.2	1.0-1.2	6	9	20.5	32
**13**	B-775	*Pseudomonas stutzeri*	0.8-0.9	1.6-1.8	1.6-1.8	1.6-1.8	12	28	48	68
**14**	B-59347	*Stenotrophomonas rhizophila*	0.7-0.8	1.5-1.6	1.5-1.6	1.5-1.6	7	12	24	36
**15**	B-787	*Lactobacillus plantarum*	1.3-1.5	2.5-2.6	2.5-2.6	2.5-2.6	10	19	41	63
**16**	B-59838	*Escherichia coli serotype O157:H7*	0.7-0.8	1.5-1.6	1.5-1.6	1.5-1.6	6	10	19	28
**17**	B-41609	*Mesorhizobium amorphae*	0.6-0.8	1.1-1.3	1.1-1.3	1.1-1.3	13	36	78	120
**18**	B-14838	*Xanthobacter flavus*	0.7-0.8	1.3-1.4	1.3-1.4	1.3-1.4	10	32	81	130
**19**	B-14393	*Bacillus amyloliquefaciens*	0.4-0.5	0.9-1.1	0.9-1.1	0.9-1.1	6	10	20	30

**20**	BD-147	*Bacillus pumilus*	1.1-1.2	2.0-2.1	2.0-2.1	2.0-2.1	10	13	26	39
**21**	B-16357	*Streptomyces atrovirens*	0.6-0.65	0.8-0.85	0.8-0.85	0.8-0.85	36	48	104	160
**22**	B-1574	*Rhodococcus erythropolis*	0.5-0.55	0.65-0.7	0.65-0.7	0.65-0.7	37	50	108	166
**23**	B-14799	*Bacillus mycoides*	0.7-0.8	1.3-1.45	1.3-1.45	1.3-1.45	16	30	95	160
**24**	B-14811	*Bacillus mycoides Flugge 1886*	0.5-0.6	1.1-1.2	1.1-1.2	1.1-1.2	6	12	76	140
**25**	B-348	*Bacillus mycoides*	0.8-0.85	1.2-1.44	1.2-1.44	1.2-1.44	17	55	107.5	160
**26**	NRS-273	*Bacillus mycoides Flugge 1886*	0.55-0.65	1.2-1.25	1.2-1.25	1.2-1.25	6	23	71.5	120
**27**	B-617	*Bacillus pseudomycoides*	0.65-0.8	1.4-1.6	1.4-1.6	1.4-1.6	10	18	81.5	145
**28**	B-14733	*Flavobacterium johnsonae*	0.5-0.6	1.1-1.2	1.1-1.2	1.1-1.2	44	72	97	122
**29**	B-14842	*Flavobacterium aquatile*	0.55-0.65	1.0-1.1	1.0-1.1	1.0-1.1	10	19	64.5	110
**30**	B-2648	*Flavobacterium resinovorum*	0.3-0.38	0.55-0.6	0.55-0.6	0.55-0.6	11	22	81	140
**31**	B-59409	*Enterobacter soli*	0.7-0.8	1.55-1.65	1.55-1.65	1.55-1.65	12	22	66	110
**32**	B-14690	*Cupriavidus necator*	0.5-0.6	1.3-1.4	1.3-1.4	1.3-1.4	7	23	74	125
**33**	B-3159	*Arthrobacter ramosus*	0.8-0.85	1.4-1.5	1.4-1.5	1.4-1.5	14	42	81	120
**34**	B-3157	*Nocardioides simplex*	0.5-0.6	1.0-1.1	1.0-1.1	1.0-1.1	11	32	77	122

**35**	B-3704	*Escherichia coli*	1.1-1.2	1.95-2.05	1.95-2.05	1.95-2.05	68	105	135	165
**36**	B-23264	*Enterobacter cloacae*	0.75-0.85	1.7-1.9	1.7-1.9	1.7-1.9	12	28	86.5	145

Note: The growth stage is divided into 4 time points. The first time point is the midpoint of the exponential phase (Exp), a crucial period as it represents a phase of active growth and metabolic activity. Since the prevalence of environmental strains predominantly residing in the stationary phase, we also included three key points within this phase: the onset of the stationary phase (S1), the midpoint (S2), and the conclusion (S3) of the stationary phase.

Table S8: Comparison of Energy Density per Unit Area between our group and published reports

Term	Abbreviation	Unit	Formula	Our Group	Setting 1 [11]	Setting 2 [11]
**Numerical Aperture**	NA		Constant	0.5	1.2	1.2
**Laser Wavelength**	λ	μm	Constant	0.532	0.532	0.532
**Spot Diameter**	d	μm	Eqn. S1	1.29808	0.54086667	0.54086667
**Spot Area**	A	μm^2	Eqn. S2	1.32273417	0.22964135	0.22964135
**Laser Intensity**	P	mW	Constant	20.93	15	100
**Power Density**	I	$mW/\mu m^2$	Eqn. S3	15.8232851	65.3192468	435.461646
**Single Scan Time**	t	s	Constant	20	5	0.1-5
**Energy Density per Unit Area**	E	$mJ/\mu m^2$	Eqn. S4	316.465703	326.596234	43.5462-2177.3082
**Reported Damages on Cell**	As shown in **Fig. S7**, we excluded the damaged cells (<5% of total measurements for pure-cultured cells), thus no damaged cells were preserved in the dataset. Damaged cells can be identified by characteristic D-band and G-band carbon peaks, which result from the removal of hydrogen and oxygen (as water vapor) and the retention of carbon as reported by the amorphous carbon spectra [12]. These intense carbon signals would overshadow the amide I peak at 1650 cm^{-1}, the key biomarker used to distinguish cells from non-cell particles. Thus, if the laser were intense enough to damage a cell, the resulting spectrum would no longer be recognized as cellular and would be excluded from the dataset.				No significant photophoretic damages were detected on exposure to 100mW or 15mW laser for SCRS measurements	

Text S1 (continued)

SCRS acquisition. Samples were first diluted and homogenized, then mounted on an optically polished CaF_2 slide (Crystran Ltd., Dorset, U. K.) before SCRS acquisition. All spectra were acquired using a Confocal Raman Microscope LabRAM HR Evolution (HORIBA France SAS), with 532nm excitation wavelength and a 50x/0.50 N.A. magnification objective (LMPIanFL N, Olympus Corporation, Tokyo, Japan). The nominal laser power was measured to be approximately 83.7 mW using laser power meter (THORLABS Digital Console with Photodiode Sensor PM121D), without a neutral density filter with 532nm excitation wavelength and a 50x/0.50 N.A. magnification objective. However, during measurements of pure-cultured and environmental cells, a 25% neutral density filter was used, reducing the actual laser power to approximately 20.9 mW. Spectra were collected with 20s acquisition time, 3 times of accumulation and 600 grating (500nm).

To facilitate comparison with previously reported studies, we calculated the energy density per unit area using **Eqn. S1–S4**. As shown in **Table S8**, our calculated energy density aligns well with previously reported values that did not induce significant photophoretic damage to cells. Furthermore, we observed no SCRS signatures indicative of burnt cells in our dataset (**Fig. S8 and Table S8**). These findings confirm that the laser parameters used in our experiment are consistent with established SCRS guidelines, ensuring that the cells remain structurally intact and unaltered [11, 13].

$$d = \frac{1.22 \cdot \lambda}{NA}, \tag{S1}$$

$$A = \pi \cdot \left(\frac{d}{2}\right)^2, \tag{S2}$$

$$I = \frac{P}{A}, \tag{S3}$$

$$E = I \cdot t, \tag{S4}$$

Where NA is the numerical aperture of the objective, λ is the laser wavelength (unit, μm), d is the laser spot diameter (unit, μm), A is the spot area (unit, μm^2), P is the laser power intensity (unit, mW), I is the laser density (unit, $mW/\mu m^2$), t is the scanning time (unit, s), E is energy density per unit area (unit, $mJ/\mu m^2$).

SCRS data processing. About 100 SCRS were collected and analyzed per strain per sample time point, resulting in a total of 17208-spectra dataset. The wavenumber range of 400-1800 cm^{-1} to was used for downstream analysis regarding as the "fingerprint" range for microorganism biomass [14]. Spectral data preprocessing includes smoothing (with Savitzky-Golay algorithm with size of 5 and degree of 3) and baseline correction (with 10-degree polynomial, max points of 256 and noise points of 64).

SCRS Signal-to-Noise Ratio (SNR) analysis. To compute the SNR for SCRS spectra, we first identify the baseline region (a range of wavenumbers under silent regions of Raman signal, here, we picked 1800-2000 cm^{-1}) to estimate the noise level. The noise is calculated as the standard deviation (σ) of the signal in this baseline region using **Eqn. S5**. Next, we determine the peak regions (ranges of wavenumbers corresponding to Raman peaks, here, we picked Amide I and phenylalanine at 995-1010 cm^{-1} and 1640-1670 cm^{-1} as we used them to identify a cell and calculate the peak signal (S_{peak}) as the mean intensity within these regions using **Eqn. S6** [15]. The SNR is then computed using **Eqn. S7**. The example spectra and SNR for the entire dataset were shown in **Fig. S7**. Within the dataset, 9.7% of the samples exhibit an SNR < 5 and 90.3%, achieve an SNR > 5, which is generally considered acceptable for machine learning classification [16].

$$\sigma_{baseline} = \sqrt{\frac{1}{N}\sum_{i=N}^{N}(I_i - \bar{I})^2}, \qquad \text{(S5)}$$

$$S_{peak} = \frac{1}{M}\sum_{j=1}^{M} I_j, \tag{S6}$$

$$SNR = \frac{Peak\ Signal}{Baseline\ Noise} = \frac{S_{peak}}{\sigma_{baseline}}, \tag{S7}$$

Where I_j represents intensities in the peak region and M is the number of points in the peak region. I_i is the intensity at a wavenumber in the baseline region, $\bar{I}$ is the mean intensity, and N is the number of points in the baseline region. If $\sigma_{baseline} = 0$, SNR is undefined.

Raman-FISH analysis. Raman-FISH combines fluorescence in situ hybridization (FISH) and Raman spectroscopy to identify functionally significant microorganisms, such as *Tetrasphaera* and *Comamonadaceae*, in enhanced biological phosphorus removal (EBPR) systems. The FISH protocol was conducted according to the previous studies without the addition of sodium dodecyl sulfate to preserve intracellular storage compounds and reduce biomass loss [17]. For *Tetrasphaera*, probes such as Tet1-266 (sequence: CCCGTCGTCGCCTGTAGC, formamide concentration: 25%), Tet2-831 (sequence: TCGTGAAATGAGTCCCAC, 10%), Tet2-842 (sequence: GCGGCACAGAACTCGTGA, 30%), and Tet3-654 (sequence: GGTCTCCCCTACCATACT, 35%) are used. For *Comamonadaceae*, the probe Cte (sequence: TTCCATCCCCCTCTGCCG, formamide concentration: 20%) is employed [18]. Fluorescence signals from these probes are observed without mounting media, and specific cells are identified and marked using LabSpec software for Raman analysis. This technique effectively integrates molecular and spectral methods for microbial identification. This technique effectively integrates molecular and spectral methods for microbial identification. More details of SCRS sample preparation and SCRS data acquisition can be found in our previous publications [18–20].

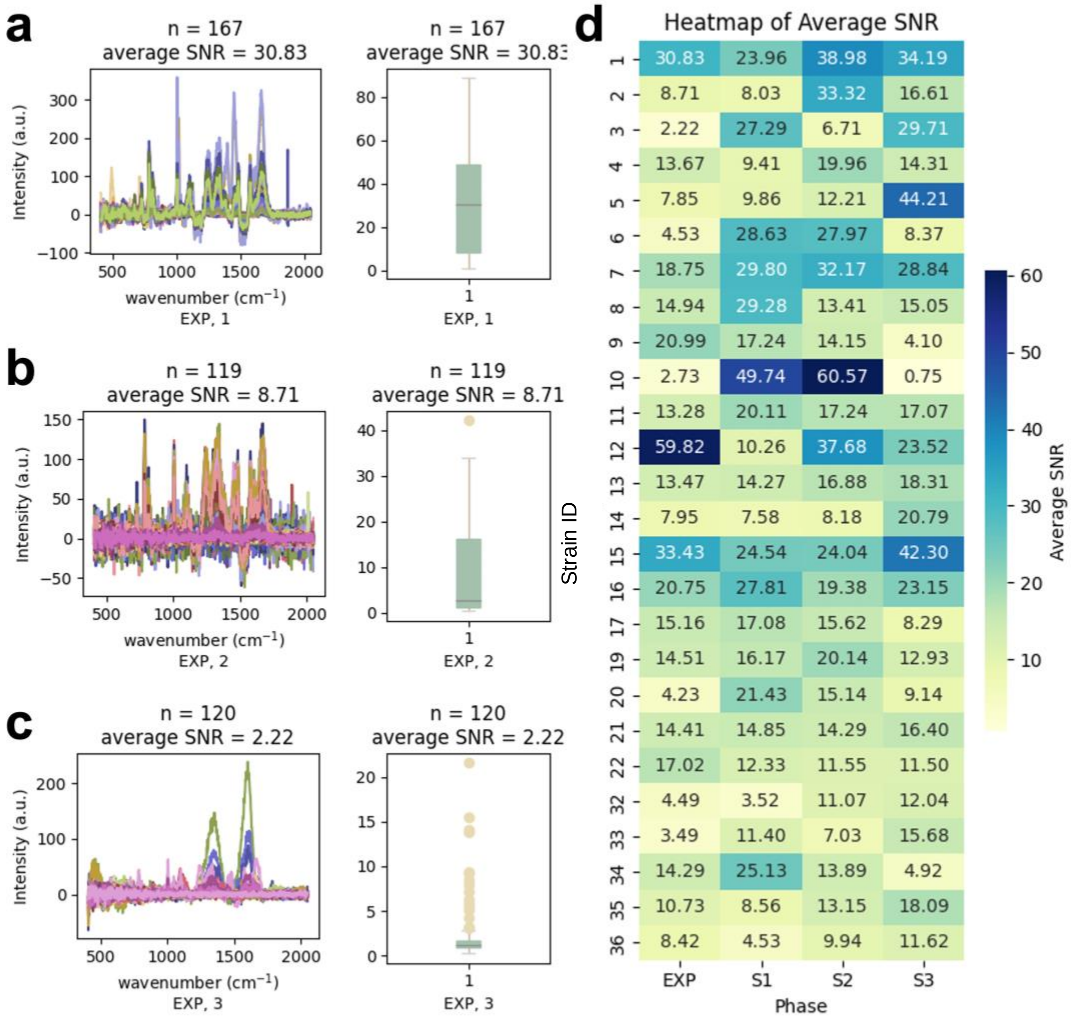

Fig. S7: Signal-to-Noise Ratio (SNR) analysis of Raman spectra across different experimental conditions and phases. (**a-c)** Representative Raman spectra and corresponding SNR boxplots for strain 1-3 at exponential phase (strain ID refers to the strain ID in **Table S1**). Left panels show overlaid Raman spectra from samples within each experiment, where n denotes the number of spectra analyzed. The right panels show boxplots of the SNR distributions. **(d)** Heatmap of average SNR values across samples (strain ID refers to the strain ID in **Table S1**) and phases (S1, S2, S3). The color intensity represents the average SNR, with higher values indicating better spectral quality.

Table S9 shows the results of statistical significance test between the accuracies of selected best-performing algorithms. **Fig. S8** below **Table S9** shows the assessment of statistical significance regarding the p-values contained in **Table S9**.

Table S9: Classification accuracy comparisons between classifier LR, SVM_LIN_CV, and SVM_RBF_CV accompanied by best-case dimensionality reduction methods. Statistical significance were assessed via BEST [1] and FDR thresholding [8].

Model of μ_1	μ_1	Model of μ_2	μ_2	p-value (BEST [1]) $P(\mu_1 \geq \mu_2)$
Exp/none+lr	0.956	S1/none+lr	0.984	0
Exp/none+lr	0.956	S2/none+lr	0.980	0
Exp/none+lr	0.956	S3/none+lr	0.971	0
S2/none+lr	0.980	S1/none+lr	0.984	0
S3/none+lr	0.971	S1/none+lr	0.984	0
S3/none+lr	0.971	S2/none+lr	0.980	0
Exp/none+svm_lin_cv	0.965	S1/none+svm_lin_cv	0.990	0
Exp/none+svm_lin_cv	0.965	S2/none+svm_lin_cv	0.987	0
Exp/none+svm_lin_cv	0.965	S3/none+svm_lin_cv	0.978	0
S2/none+svm_lin_cv	0.987	S1/none+svm_lin_cv	0.990	0
S3/none+svm_lin_cv	0.978	S1/none+svm_lin_cv	0.990	0
S3/none+svm_lin_cv	0.978	S2/none+svm_lin_cv	0.987	0
Exp/none+svm_rbf_cv	0.950	S1/none+svm_rbf_cv	0.983	0
Exp/none+svm_rbf_cv	0.950	S2/none+svm_rbf_cv	0.977	0
Exp/none+svm_rbf_cv	0.950	S3/none+svm_rbf_cv	0.966	0
S2/none+svm_rbf_cv	0.977	S1/none+svm_rbf_cv	0.983	0
S3/none+svm_rbf_cv	0.966	S1/none+svm_rbf_cv	0.983	0
S3/none+svm_rbf_cv	0.966	S2/none+svm_rbf_cv	0.977	0
Exp/lda+lr	0.964	S1/lda+lr	0.991	0
Exp/lda+lr	0.964	S2/lda+lr	0.985	0
Exp/lda+lr	0.964	S3/lda+lr	0.979	0
S2/lda+lr	0.985	S1/lda+lr	0.991	0
S3/lda+lr	0.979	S1/lda+lr	0.991	0
S3/lda+lr	0.979	S2/lda+lr	0.985	0
Exp/lda+svm_lin_cv	0.963	S1/lda+svm_lin_cv	0.992	0
Exp/lda+svm_lin_cv	0.963	S2/lda+svm_lin_cv	0.981	0
Exp/lda+svm_lin_cv	0.963	S3/lda+svm_lin_cv	0.973	0
S2/lda+svm_lin_cv	0.981	S1/lda+svm_lin_cv	0.992	0
S3/lda+svm_lin_cv	0.973	S1/lda+svm_lin_cv	0.992	0
S3/lda+svm_lin_cv	0.973	S2/lda+svm_lin_cv	0.981	0
Exp/lda+svm_rbf_cv	0.962	S1/lda+svm_rbf_cv	0.983	0

Exp/lda+svm_rbf_cv	0.962	S2/lda+svm_rbf_cv	0.976	0
S3/lda+svm_rbf_cv	0.953	Exp/lda+svm_rbf_cv	0.962	0
S2/lda+svm_rbf_cv	0.976	S1/lda+svm_rbf_cv	0.983	0.0001
S3/lda+svm_rbf_cv	0.953	S1/lda+svm_rbf_cv	0.983	0
S3/lda+svm_rbf_cv	0.953	S2/lda+svm_rbf_cv	0.976	0

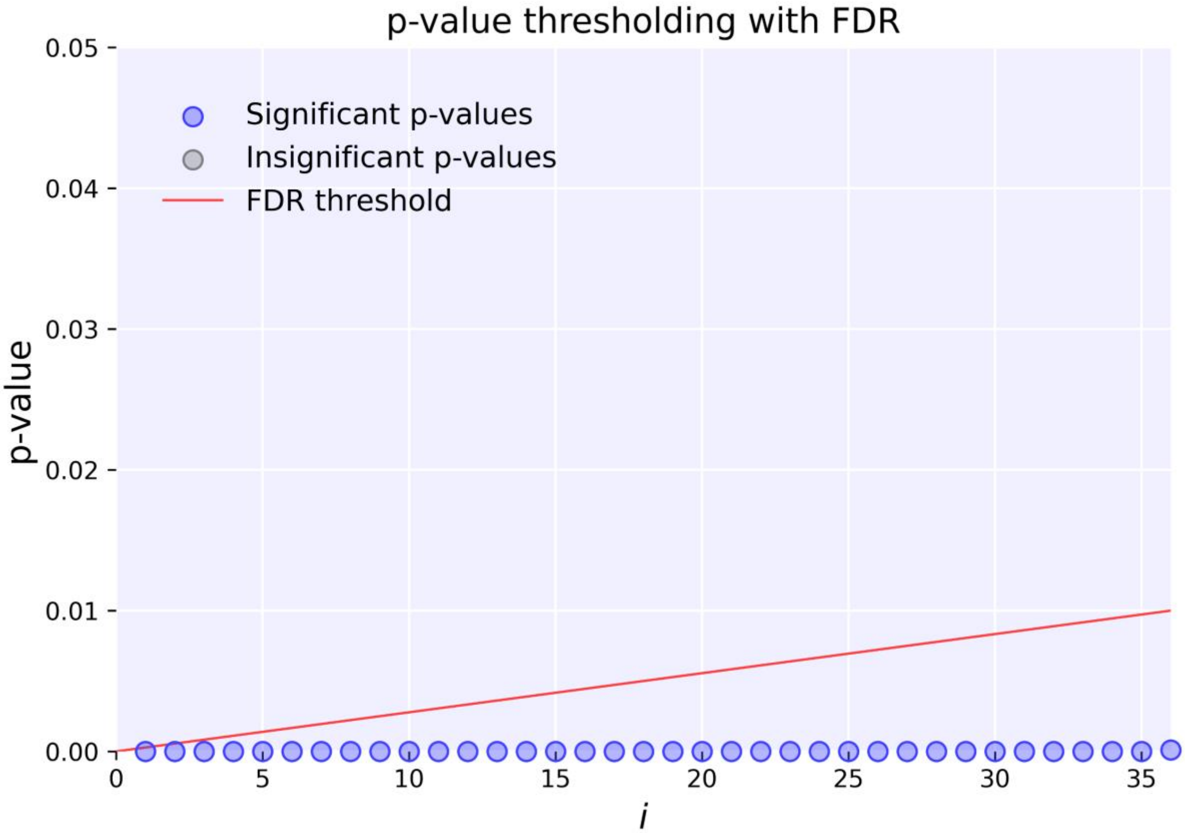

Fig. S8: P-values from the multiple null-hypothesis test in **Table S9** filtered by FDR thresholding. Significance level α=0.01. The p-values below the red threshold line are considered significant.

Text S2: DR Algorithms

Principal component analysis (PCA). PCA is arguably the most widely used dimensionality reduction method in SCRS classification. PCA is linear and unsupervised which projects original data into a subspace spanned by principal components, which represents the directions of highest variances in the original data space [6]. PCA is widely used as a DR method as it has many nice properties, including being the best approximation of the original dataset for variances [3]. As a weakness, PCA could fail when latent sources of variations in the dataset are not independent [21].

Linear discriminant analysis (LDA). LDA is a linear, supervised DR algorithm that finds the output space by selecting the directions along which samples from the same classes tend to cluster together making the class distinctive and separable from other classes [3]. LDA has a deep connection with multivariate Gaussian distribution and assumes samples in each class are normally distributed [3].

Supervised PCA (Sup-PCA). Sup-PCA is a supervised variant of canonical PCA for DR, by finding a subspace such that the projected data has maximum relevance to the labels, measured in Hilbert-Schmidt independence criterion (HSIC) [5, 22]. It can be formulated as optimizing a projection U by

$$\arg\max_{U} \mathbf{tr}(U^T X H L H X^T U) \qquad \text{s.t. } U^T U = I,$$

where $U^T X$ is projected data X, H is the centering matrix, and L is a kernel of labels [5]. In this study we use

$$L = Y^T Y,$$

where Y is the one-hot encoding of label vector y.

Kernel PCA (KPCA). KPCA is a nonlinear form of PCA with the kernel method incorporated [4]. With an appropriate kernel function, the original data can be projected into a higher dimensional

space for better capturing its nonlinear relationships than canonical PCA. In this study, the radial basis function kernel (RBF kernel) was chosen and used in KPCA, as it is arguably the most widely used kernel function that can approximate a wide range of nonlinear functions.

Iterative spectrum method supervised dimensionality reduction (ISM-SDR). ISM-SDR is a novel nonlinear supervised-DR developed recently by Wu et al. [2]. Unlike KPCA where the linear projection is after the feature map, the linear projection for ISM-SDR is prior to the feature map. The projection is identified by maximizing the dependency between the projected data and the label via HSIC. It is noticeable that ISM-SDR requires balanced data, i.e., all classes are expected to have similar proportions in the dataset. The details of ISM-SDR implementation can be found at https://github.com/albat3ross/ISM_supervised_DR.

Text S3: Classifiers

Gaussian naïve Bayes classifier (GNB) and K-nearest neighbor classifier (KNN). These two models are included for their simplicity. GNB makes decisions by finding the class with the highest posterior probability that a new sample can belong to, assuming that all features are Gaussian and independent. KNN classifies a testing sample by plurality votes from training samples closest to it. In this study, we used 10 nearest samples, i.e. parameter k = 10.

Logistic regression (LR). LR is a classical and widely used linear classifier, named after the logistic function (sigmoid function) used in its formulation [6]. In binary cases, LR has a strong relationship with GNB [23]. In this study, LR was trained in the one-versus-one strategy for multi-class classification.

Random forest (RF) classifier. RF is an ensemble of decision trees (DT). DT is a classifier model with a tree-like structure. A sample is classified by a traversal through the tree according to a hierarchy of value-based tests at each internal node, till reaching a leaf node which represents the predicted label [24]. In our setup, each DT was trained with random subsets from the training data

(known as bagging) and the final label was the majority vote from all DTs. Theoretically, an RF ensembled with more DTs always leads to lower error rates [3]. In this study, the number of decision trees is 100.

Linear discriminant analysis (LDA). In this study, LDA is the only model that can be used both as a DR and a classifier. When used as a classifier, LDA assesses the posterior probability of the testing sample being a member of each class, by identifying if it has features valued in its "signature regions" that have a high local density of training samples from a particular class. LDA assumes that all classes share the same covariance matrix, making it different from GNB. In this study, we chose a one-vs-one approach rather than the one-vs-rest for multi-class LDA, as the latter will result in significantly imbalanced data in our case which is believed to hinder LDA performance [25].

Support vector machine (SVM) and kernel support vector machine (KSVM). SVM is a linear classifier featured with a linear decision boundary that maximizes the margin between two classes [7]. The sample points from both classes that are closest to the decision boundary are called support vectors. SVM is known to be effective in working with high dimensional data (e.g. SCRS) as it is intrinsically regularized to control overfitting [26]. Kernel support vector machine (KSVM) is a nonlinear variant of SVM incorporating kernel method [6]. In this study, we chose the RBF kernel as it is flexible to approximate a wide type of nonlinear functions. The SVM and KSVM are implemented as C-SVM featuring a regularization parameter C. SVM_LIN and SVM_RBF used its default value (C = 1.0), while SVM_LIN_CV and SVM_RBF_CV had it optimized via 10-fold cross-validation.

Multi-layer Perceptron (MLP). MLP is one type of Neural Network (NN) that can be used as a versatile nonlinear classifier inspired by the biological connections between neurons. MLP is commonly trained by stochastic gradient descent, where in each iteration the weights are perturbed to decrease the objective (loss) of a subset of the training data. In this study, we deployed a 4-layer network with a width of 2000 neurons using Cross-Entropy as the objective.

Text S4: Traditional Classifiers Perform as Well as Neural Network with Better Explainability

Neural network (NN) is an emerging model type that has achieved impressive results in advanced learning tasks. Here we also tried MLP (a type of NN) and compared it with the tested traditional classifiers. LDA+NN had marginal accuracy differences from traditional classifiers in both T-only and T/G setups and was not always the outperformer (**Figs. S9a&b**), though differences can still be statistically significant (p-value < 0.001) (**Fig. S9c**). These on-par performances indicate that the underlying relationship in our data is linear, thus the nonlinearity necessitating the employment of NN is limited. Therefore, we suggest that NN is not necessarily superior to traditional classifiers in this application case. NN also showed some disadvantages in comparison to traditional classifiers. During out testing, signs of overfitting were noticed on NN for (nearly) perfect error rate (< 0.2%) training accuracies given the testing error rate being a magnitude bigger (1.1%-3.7%). Considering that our training dataset size (90% of 17,208) is below conventional neural network requirements (e.g. number of input dimensionality squared, 1024^2), this overfitting could potentially be caused suboptimal training data size. The slow training was another disadvantage, being observed 5 - 10x longer in wall clock time than SVMs (with CV parameter optimization), and 100 - 5000x longer compared to other simple and explainable models such as LR (data not shown). Note that traditional models are also easier to deploy as they do not require GPUs to achieve an acceptable level of time efficiency. While we suggest further investigations on larger datasets in the future, traditional classifiers such as SVM and LR will likely suffice SCRS datasets sized similar to ours.

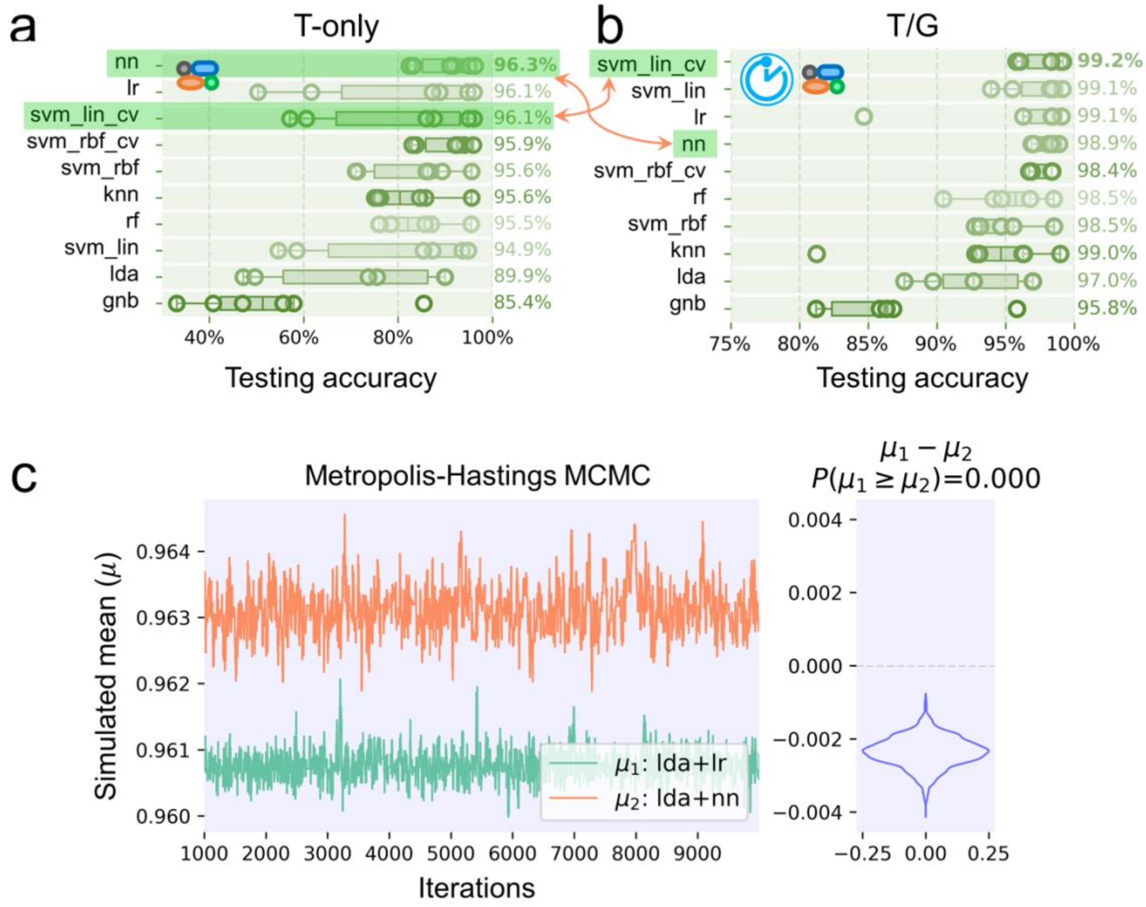

Fig. S9: Taxonomy classification accuracy comparison between classifiers, with neural networks (NN) results included. **(a)** NN had a 0.2% margin of outperformances over the best traditional classifier in the T-only approach, and **(b)** a 0.3% margin of underperformance in the T/G approach. Overall, traditional classifiers can perform as competitive as NN in taxonomy classification, though the situational outperformance could be statistically significant as shown in **(c)** (p-value < 0.001).

Text S5: ML-Enabled SCRS Offers Molecular Insights into Microbial Structures and Functional Profiles.

We conducted a feature ranking analysis of SCRS spectra to gain molecular insights into microbial structures and functional profiles, in varying growth stages. **Figs. S10a&b** showed the features

(i.e., SCRS wavenumbers revealed chemical bonds) ranked by their ability to differentiate growth stages within each taxonomy and taxonomies within each growth stage, respectively. The Laplacian score measures how well spectra from the same label cluster together along a specific feature [27]. Our results indicated that proteins and lipids consistently received high-ranking scores across all strains, overlapping with bands that had greater power to differentiate cell stages. This overlap may partially explain why the G-T model exhibited lower accuracy compared to the taxonomy-only model. Further comparative analysis identified several SCRS peaks with high rankings that contributed to differentiating taxonomy from growth stages, including tyrosine (855 cm^{-1}), carbohydrates (1042 cm^{-1}), glucose (1125 cm^{-1}), and pyridyl (1601 cm^{-1}) [15]. These findings help pinpoint specific biochemicals or metabolites related to strain and growth stage differences, revealing their detailed metabolic traits. While more in-depth and comprehensive feature ranking and identification analysis is warranted in future studies, this study underscores that machine learning-enabled SCRS can effectively provide molecular insights into microbial structures and functional profiles.

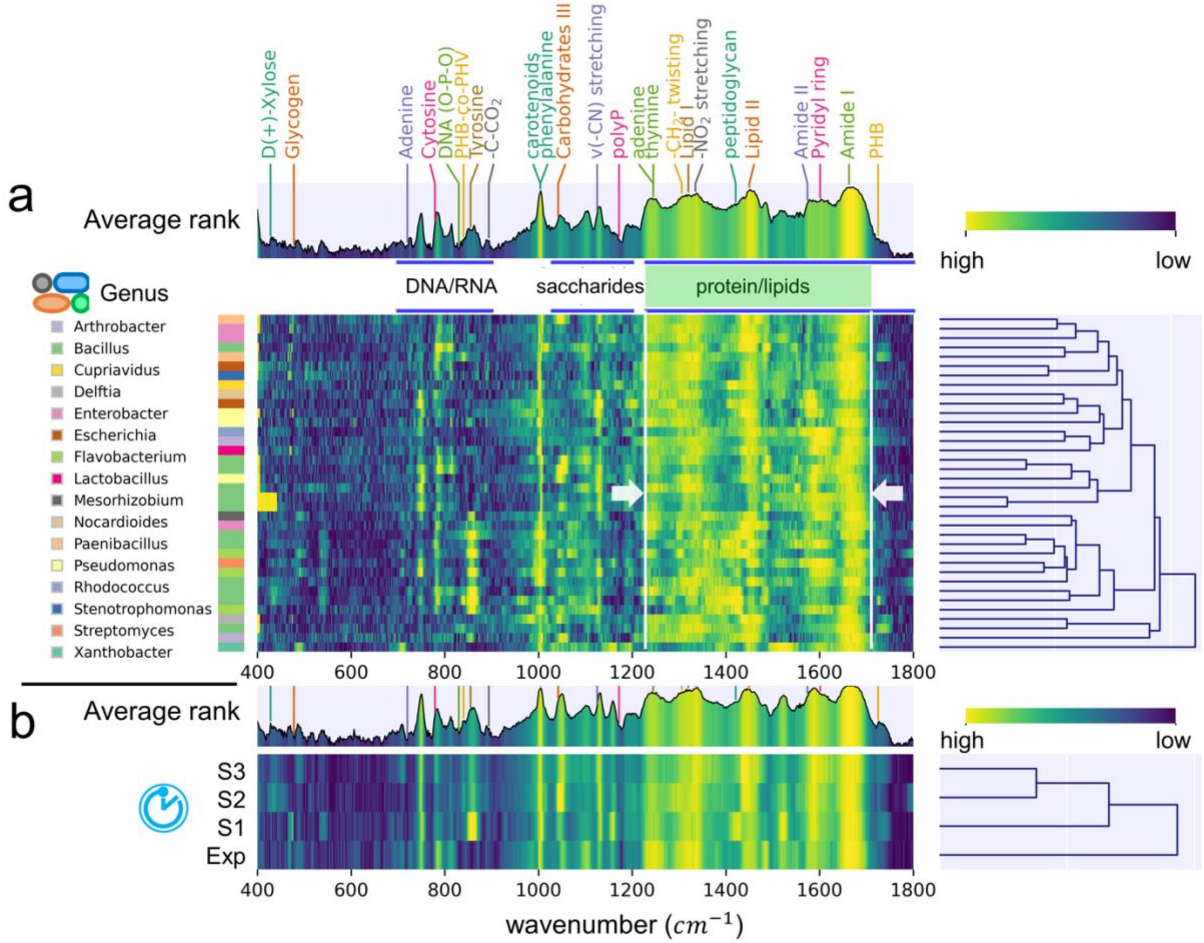

Fig. S10: Feature ranking of SCRS profiles of 36 strains by Laplacian score [27]: **(a)** distinguishable SCRS-fingerprints associated with cells of each taxonomy at genus level; **(b)** distinguishable SCRS-fingerprints associated with cells from four distinct growth stages. The curve on top of each heatmap shows the average ranking.

REFERENCES

1. Kruschke JK. Bayesian estimation supersedes the t test. *J Exp Psychol Gen* 2013; **142**: 573.
2. Wu C, Miller J, Chang Y, Sznaier M, Dy J. Solving interpretable kernel dimensionality reduction. *Adv Neural Inf Process Syst* 2019; **32**.

3. Hastie T, Tibshirani R, Friedman JH, Friedman JH. The elements of statistical learning: data mining, inference, and prediction. 2009. Springer.
4. Schölkopf B, Smola A, Müller K-R. Nonlinear Component Analysis as a Kernel Eigenvalue Problem. *Neural Comput* 1998; **10**: 1299–1319.
5. Barshan E, Ghodsi A, Azimifar Z, Jahromi MZ. Supervised principal component analysis: Visualization, classification and regression on subspaces and submanifolds. *Pattern Recognit* 2011; **44**: 1357–1371.
6. Mohri M, Rostamizadeh A, Talwalkar A. Foundations of machine learning. 2018. MIT press.
7. Boser BE, Guyon IM, Vapnik VN. A training algorithm for optimal margin classifiers. *Proc. 5th Annu. ACM Workshop Comput. Learn. Theory*. pp 144–152.
8. Benjamini Y, Hochberg Y. Controlling the False Discovery Rate: A Practical and Powerful Approach to Multiple Testing. *J R Stat Soc Ser B Methodol* 1995; **57**: 289–300.
9. Welcome to the ARS Culture Collection (NRRL) | ARS Culture Collection. https://nrrl.ncaur.usda.gov/. Accessed 17 Dec 2024.
10. Li G, Wu C, Wang D, Srinivasan V, Kaeli DR, Dy JG, et al. Machine Learning-Based Determination of Sampling Depth for Complex Environmental Systems: Case Study with Single-Cell Raman Spectroscopy Data in EBPR Systems. *Environ Sci Technol* 2022; **56**: 13473–13484.
11. Lee KS, Palatinszky M, Pereira FC, Nguyen J, Fernandez VI, Mueller AJ, et al. An automated Raman-based platform for the sorting of live cells by functional properties. *Nat Microbiol* 2019; **4**: 1035–1048.

12. Dychalska A, Popielarski P, Franków W, Fabisiak K, Paprocki K, Szybowicz M. Study of CVD diamond layers with amorphous carbon admixture by Raman scattering spectroscopy. *Mater Sci-Pol* 2015; **33**: 799–805.

13. Butler HJ, Ashton L, Bird B, Cinque G, Curtis K, Dorney J, et al. Using Raman spectroscopy to characterize biological materials. *Nat Protoc* 2016; **11**: 664–687.

14. Wang D, He P, Wang Z, Li G, Majed N, Gu AZ. Advances in single cell Raman spectroscopy technologies for biological and environmental applications. *Curr Opin Biotechnol* 2020; **64**: 218–229.

15. Golparvar A, Boukhayma A, Enz CC, Carrara S. Optimized Detection of Hypoglycemic Glucose Ranges in Human Serum by Raman Spectroscopy with 532 nm Laser Excitation. *PHOTOPTICS*. 2022. pp 158–165.

16. Thomsen BL, Christensen JB, Rodenko O, Usenov I, Grønnemose RB, Andersen TE, et al. Accurate and fast identification of minimally prepared bacteria phenotypes using Raman spectroscopy assisted by machine learning. *Sci Rep* 2022; **12**: 16436.

17. Fernando EY, McIlroy SJ, Nierychlo M, Herbst F-A, Petriglieri F, Schmid MC, et al. Resolving the individual contribution of key microbial populations to enhanced biological phosphorus removal with Raman–FISH. *ISME J* 2019; **13**: 1933–1946.

18. Yan Y, Han I, Lee J, Li G, Srinivasan V, McCullough K, et al. Revisiting the role of Acinetobacter spp. in side-stream enhanced biological phosphorus removal (S2EBPR) systems. *Water Res* 2024; **251**: 121089.

19. Onnis-Hayden A, Srinivasan V, Tooker NB, Li G, Wang D, Barnard JL, et al. Survey of full-scale sidestream enhanced biological phosphorus removal (S2EBPR) systems and comparison with conventional EBPRs in North America: Process stability, kinetics, and microbial populations. *Water Environ Res* 2020; **92**: 403–417.

20. Wang D, Li Y, Cope HA, Li X, He P, Liu C, et al. Intracellular polyphosphate length characterization in polyphosphate accumulating microorganisms (PAOs): Implications in PAO phenotypic diversity and enhanced biological phosphorus removal performance. *Water Res* 2021; **206**: 117726.

21. Shlens J. A tutorial on principal component analysis. *ArXiv Prepr ArXiv14041100* 2014.

22. Gretton A, Bousquet O, Smola A, Schölkopf B. Measuring Statistical Dependence with Hilbert-Schmidt Norms. In: Simon HU, Tomita E (eds).2005. Springer Berlin Heidelberg, Berlin, Heidelberg, pp 63–77.

23. Mitchell T. Machine Learning. 1997. McGraw-Hill Education.

24. Breiman L, Friedman JH, Olshen RA, Stone CJ. Classification and regression trees. 1984. Routledge.

25. Xie J, Qiu Z. The effect of imbalanced data sets on LDA: A theoretical and empirical analysis. *Pattern Recognit* 2007; **40**: 557–562.

26. Zhou Q, Chen W, Song S, Gardner J, Weinberger K, Chen Y. A reduction of the elastic net to support vector machines with an application to GPU computing. *Proc. AAAI Conf. Artif. Intell.* 2015.

27. He X, Cai D, Niyogi P. Laplacian score for feature selection. *Adv Neural Inf Process Syst* 2005; **18**.
